# Narciclasine Exerts Anticancer Activity in Colorectal Cancer Cells in Association with HELLS Downregulation and DNA Damage-Associated Responses

**DOI:** 10.3390/ph19091334

**Published:** 2026-08-24

**Authors:** Yoon-Mi Lee, Sumin Han, Gyun Seok Park, Judy Gopal, Jae-Wook Oh

**Affiliations:** 1 Department of Stem Cell and Regenerative Biotechnology, Konkuk University, 120 Neungdong-ro, Gwangjin-gu, Seoul 05029, Republic of Korea; dldbsal1414@konkuk.ac.kr (Y.-M.L.); hsm534@naver.com (S.H.);; 2 Department of Research and Innovation, Saveetha School of Engineering, Saveetha Institute of Medical and Technical Sciences (SIMATS), Thandalam, Chennai 602105, India; jejudy777@gmail.com

**Keywords:** narciclasine, *Lycoris sanguinea*, colorectal cancer, HELLS, DNA damage response, G2/M arrest, apoptosis

## Abstract

**Background/Objectives:** Colorectal cancer (CRC) remains a major cause of cancer-related mortality worldwide, largely owing to metastasis, recurrence, and resistance to conventional therapies. Natural-product-derived compounds represent an important source of anticancer drug candidates. This study investigated the anticancer activity of purified narciclasine, which was tentatively annotated as a major detected constituent of *Lycoris sanguinea* extract, and examined its association with HELLS-associated DNA damage responses in CRC cells. **Methods:** Human CRC cell lines HT29 and HCT116 were used to evaluate the cytotoxic and mechanistic effects of *L. sanguinea* extract and narciclasine. Cell viability, clonogenic growth, apoptosis, mitochondrial membrane potential, cell cycle distribution, migration, and invasion were assessed. Western blotting was performed to analyze apoptosis-, cell cycle-, epithelial–mesenchymal transition (EMT)-, and DNA damage-related proteins. UPLC-QTOF/MS analysis was used to identify major phytochemical constituents. HELLS knockdown and γ-H2AX immunofluorescence staining were conducted to examine the involvement of HELLS in narciclasine-associated DNA damage and apoptosis. **Results:** *L. sanguinea* extract reduced CRC cell viability, suppressed clonogenic growth, induced mitochondrial dysfunction and caspase-dependent apoptosis, and promoted G2/M cell cycle arrest with decreased cyclin B1 and increased p53/p21 expression. The extract also inhibited migration and invasion, accompanied by reduced β-catenin, N-cadherin, vimentin, slug, and snail expression and increased E-cadherin expression. UPLC-QTOF/MS tentatively annotated narciclasine as a major detected constituent. Purified narciclasine recapitulated several anticancer effects of the extract, accompanied by HELLS downregulation, increased γ-H2AX accumulation, reduced cyclin B1 expression, and enhanced PARP and caspase-3 cleavage. HELLS knockdown further sensitized CRC cells to narciclasine-associated γ-H2AX accumulation, G2/M arrest-associated signaling, and apoptosis. **Conclusions:** Narciclasine treatment was associated with HELLS downregulation, DNA damage-associated signaling, G2/M cell cycle arrest-associated changes, EMT suppression, and apoptosis in CRC cells. These findings suggest that narciclasine is a promising natural-product-derived lead compound for further preclinical evaluation in HELLS-associated CRC vulnerabilities.

## 1. Introduction

Colorectal cancer (CRC) remains one of the most prevalent and lethal malignancies worldwide. According to recent global cancer estimates, more than 1.9 million new cases of CRC and approximately 904,000 CRC-related deaths occurred in 2022, ranking CRC among the leading causes of cancer incidence and mortality [1]. Although advances in surgical resection, cytotoxic chemotherapy, targeted therapy, and immune checkpoint blockade have improved clinical outcomes, therapeutic failure remains common in patients with advanced or metastatic disease. Standard regimens based on fluoropyrimidines, oxaliplatin, irinotecan, and targeted agents against epidermal growth factor receptor (EGFR) or vascular endothelial growth factor (VEGF) signaling are frequently limited by toxicity, tumor heterogeneity, and intrinsic or acquired resistance [2,3]. Moreover, immune checkpoint inhibitors are highly effective only in a restricted subset of patients with mismatch repair-deficient or microsatellite instability-high tumors [4,5]. These limitations highlight the need for novel anticancer agents that target molecular vulnerabilities involved in CRC progression, DNA damage tolerance, and metastatic behavior.

Natural products have long served as a major source of anticancer drug discovery. Several clinically used anticancer agents, including paclitaxel and camptothecin derivatives, originated from plant-derived bioactive compounds and exert their pharmacological effects by targeting essential processes such as mitosis and DNA topology [6,7,8,9]. These precedents support the continued exploration of phytochemicals as structurally diverse scaffolds for the development of new anticancer therapeutics. Among natural-product-rich plant families, Amaryllidaceae species are known to produce biologically active alkaloids with cytotoxic, anti-inflammatory, and neuropharmacological activities [10]. The genus *Lycoris*, in particular, contains multiple alkaloids with reported anticancer potential, including lycorine and narciclasine [11,12,13].

Narciclasine is an isocarbostyril alkaloid originally characterized from Amaryllidaceae plants and has been reported to exhibit potent anticancer activity across several tumor types. Previous studies have shown that narciclasine suppresses cancer cell proliferation by interfering with protein synthesis, inducing cell cycle arrest, promoting apoptosis, and modulating signaling pathways associated with tumor growth and inflammation [14,15,16,17]. Recent evidence further suggests that narciclasine may act as a topoisomerase I inhibitor and exert cytotoxic effects through DNA damage-associated mechanisms [11,13]. However, the molecular determinants that mediate narciclasine sensitivity in CRC cells remain incompletely defined. In particular, whether narciclasine affects chromatin remodeling factors that regulate DNA damage tolerance and cell cycle progression has not been fully investigated.

*Lycoris sanguinea* is an East Asian *Lycoris* species that remains less extensively studied than other species such as *L. radiata* and *L. aurea*. Previous phytochemical studies have identified alkaloids and related compounds from *L. sanguinea*, but its anticancer potential and active constituents remain insufficiently characterized [18,19]. Therefore, chemical profiling and biological evaluation of *L. sanguinea* may provide useful insight into natural-product-derived anticancer candidates. In this study, we first evaluated the anticancer effects of *L. sanguinea* extract in CRC cells and then used ultra-performance liquid chromatography–quadrupole time-of-flight mass spectrometry (UPLC-QTOF/MS) to tentatively annotate narciclasine as a major detected constituent. We further examined whether purified narciclasine recapitulates the biological effects of the extract and investigated its associated molecular mechanism.

Helicase lymphoid-specific protein (HELLS, also known as LSH or SMARCA6) is an ATP-dependent chromatin remodeler implicated in DNA replication, chromatin accessibility, DNA repair, epigenetic regulation, and cancer progression [20,21,22]. HELLS contributes to replication fork stability and genome maintenance, and its dysregulation has been associated with increased proliferative capacity, DNA damage tolerance, and malignant progression in several cancers [23,24,25]. In CRC, HELLS has been reported to be upregulated and functionally linked to cell proliferation, cell cycle progression, and poor clinicopathological features [21,26,27]. Because HELLS supports DNA repair and chromatin remodeling programs that may allow cancer cells to tolerate genotoxic stress, it represents a potential molecular vulnerability for anticancer intervention.

In the present study, we investigated the anticancer activity of *L. sanguinea* extract and narciclasine in human CRC cell lines HT29 and HCT116, with a particular focus on HELLS-associated DNA damage responses. We show that *L. sanguinea* extract suppresses CRC cell viability, clonogenic growth, migration, and invasion while inducing mitochondrial dysfunction, G2/M cell cycle arrest, and apoptosis. UPLC-QTOF/MS analysis tentatively annotated narciclasine as a major detected constituent of the extract, and purified narciclasine recapitulated the anticancer effects of the extract. At the molecular level, narciclasine treatment was associated with reduced HELLS expression, increased γ-H2AX accumulation, reduced cyclin B1 expression, and enhanced PARP and caspase-3 cleavage. Furthermore, HELLS knockdown sensitized CRC cells to narciclasine-associated DNA damage signaling and apoptosis. These findings suggest that narciclasine exerts anticancer activity in CRC cells in association with HELLS downregulation and support further investigation of HELLS-associated DNA damage vulnerabilities in CRC.

## 2. Results

### 2.1. Lycoris sanguinea Extract Induces Apoptosis and Mitochondrial Membrane Disruption in CRC Cells

**Figure 1:**
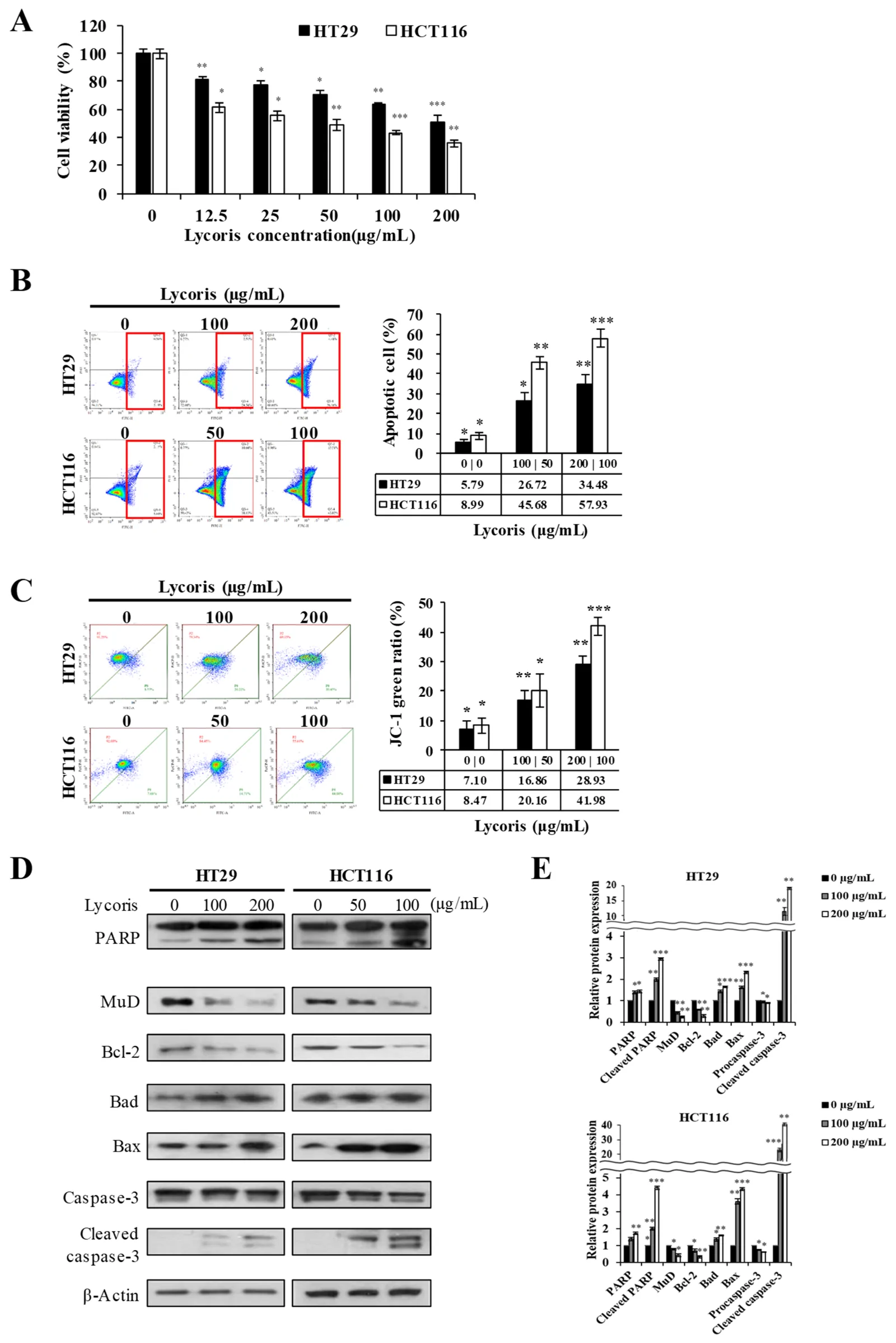
Apoptosis and mitochondrial dysfunction induced by *Lycoris sanguinea* extract in CRC cells. (**A**) Cell viability of HT29 and HCT116 cells treated with increasing concentrations of *L. sanguinea* extract for 24 h, assessed by CCK-8 assay. (**B**) Flow cytometry analysis of apoptotic cells after 24 h treatment. (**C**) Flow cytometry analysis of mitochondrial membrane potential (ΔΨm) disruption following 24 h treatment. (**D**) Western blot analysis of apoptosis-related proteins after 24 h treatment. (**E**) Densitometric quantification of the apoptosis-related proteins shown in panel (**D**), normalized to β-actin. Data are presented as mean ± SD from three independent experiments. Statistical significance: * *p* < 0.05, ** *p* < 0.01, *** *p* < 0.001 versus control.

To evaluate the cytotoxicity of *Lycoris sanguinea* extract on CRC cell lines, cell viability assays were performed on HT29 and HCT116 cells in a dose-dependent manner for 24 h. Cell viability decreased significantly in both cell lines, with HT29 cells showing 61.6 ± 1.54% and 52.38 ± 4.58% viability at 100 and 200 µg/mL, respectively. HCT116 cells were more sensitive, with viabilities of 48.96 ± 4.41% at 50 µg/mL and 41.13 ± 2.18% at 100 µg/mL (Figure 1A), indicating stronger cytotoxic effects in HCT116 cells.

To determine whether cytotoxicity was mediated by apoptosis, Annexin V/PI assays and JC-1 staining were performed. In HCT116 cells, treatment with 50 and 100 µg/mL induced apoptosis in 34.48 ± 3.54% and 57.93 ± 2.41% of cells, respectively. In HT29 cells, apoptotic rates were 19.97 ± 1.69% and 24.21 ± 1.80% at 100 and 200 µg/mL (Figure 1B). JC-1 staining revealed a dose-dependent increase in green-positive cells, indicating loss of ΔΨm. Green fluorescence increased to 33.41 ± 2.29% and 41.98 ± 2.03% in HCT116, and to 21.62 ± 1.74% and 28.93 ± 2.51% in HT29 cells at the respective concentrations (Figure 1C).

Western blot analysis was conducted to examine key apoptotic markers (Figure 1D). *L. sanguinea* extract treatment markedly increased cleaved caspase-3 and PARP levels, consistent with apoptosis induction. In HT29 cells, cleaved caspase-3 levels increased by 12.22- and 18.85-fold, and cleaved PARP by 1.39- and 1.43-fold at 100 and 200 µg/mL, respectively. In HCT116, cleaved caspase-3 levels increased by 20.96- and 37.57-fold, and cleaved PARP by 1.45- and 1.73-fold at 50 and 100 µg/mL. Pro-apoptotic proteins bax and bad were upregulated, while anti-apoptotic proteins bcl-2 and MuD were downregulated. In HT29, bax and bad increased by 1.62~2.32-fold and 1.43~1.64-fold, respectively, with bcl-2 decreasing to 0.59~0.29 and MuD to 0.44~0.25. In HCT116, bax and bad increased by 3.61~4.34-fold and 1.37~1.60-fold, with bcl-2 decreasing to 0.70~0.34 and MuD to 0.79~0.42 (Figure 1E).

These results indicate that *Lycoris sanguinea* extract induces cytotoxicity in CRC cells through apoptosis and mitochondrial dysfunction, with a more pronounced effect in HCT116 cells.

### 2.2. Lycoris sanguinea Extract Suppresses Proliferation and Induces Cell Cycle Arrest in CRC Cells

**Figure 2:**
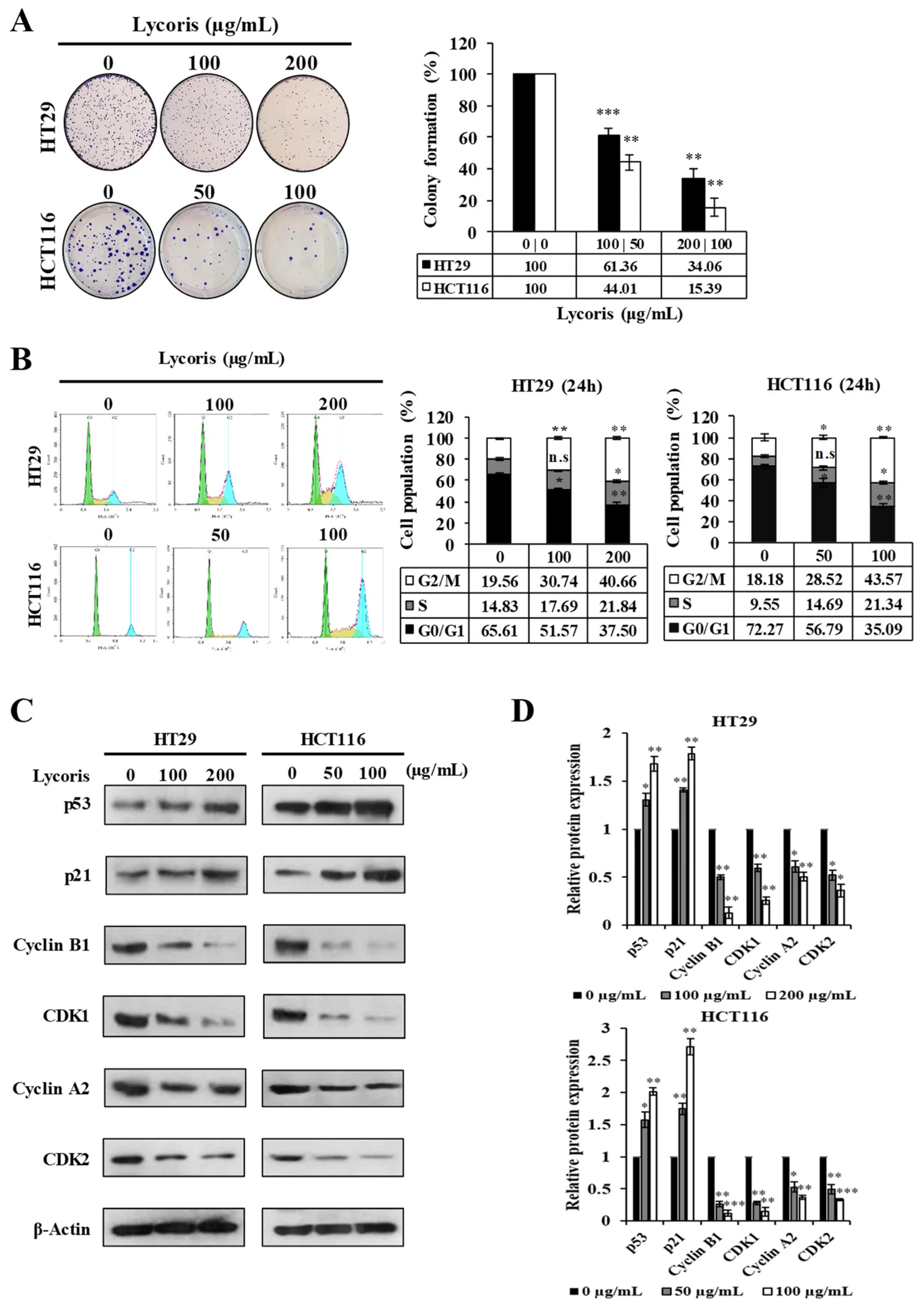
*Lycoris sanguinea* extract suppresses proliferation and induces G_2_/M phase arrest in CRC cells. (**A**) Clonogenic assay of HT29 and HCT116 cells treated with *L. sanguinea* extract for 24 h, reseeded, and cultured for 10 days. Colonies were fixed and stained with 1% crystal violet. (**B**) Flow cytometry analysis of cell cycle distribution after 24 h *L. sanguinea* extract treatment. (**C**) Western blot analysis of cell cycle regulatory proteins following 24 h treatment. (**D**) Densitometric quantification of p53, p21, cyclin B1, CDK1, cyclin A2, and CDK2 expression relative to β-actin. Data are presented as mean ± SD from three independent experiments. Statistical significance: * *p* < 0.05, ** *p* < 0.01, *** *p* < 0.001 versus control.

Given the pro-apoptotic effects of *Lycoris sanguinea* extract, we next assessed its impact on CRC cell proliferation. Clonogenic assays revealed a marked inhibition of colony-forming ability in both HT29 and HCT116 cells. At the highest concentrations, colony formation decreased to 34.06 ± 2.62% in HT29 and 15.39 ± 2.15% in HCT116 cells relative to controls (Figure 2A).

Flow cytometry analysis was performed to determine whether the anti-proliferative effect would be involved in cell cycle regulation. *Lycoris* treatment induced G_2_/M phase arrest in both cell lines. In HCT116 cells, the G_2_/M population increased from 18.18 ± 2.51% in control cells to 28.52 ± 1.89% and 43.57 ± 1.23% at 50 and 100 µg/mL, respectively. In HT29 cells, the G_2_/M population increased from 19.56 ± 1.26% to 30.74 ± 1.47% and 40.66 ± 1.81% at 100 and 200 µg/mL, respectively. A modest increase in S-phase cells was also observed, though less pronounced than G_2_/M arrest (Figure 2B).

Western blot analysis of key cell cycle regulators revealed upregulation of the tumor suppressors p53 and p21 following *Lycoris* treatment. In contrast, cyclin B1 and CDK1, critical for the G_2_/M transition, were markedly downregulated. Proteins involved in S-phase progression, including cyclin A2 and CDK2, were also reduced, albeit to a lesser extent (Figure 2C,D).

These results indicate that *Lycoris sanguinea* extract suppresses CRC cell proliferation by inducing G_2_/M phase arrest and disrupting cell cycle progression, with pronounced effects in both HT29 and HCT116 cells.

### 2.3. Lycoris sanguinea Extract Suppresses EMT in Colorectal Cancer Cells

**Figure 3:**
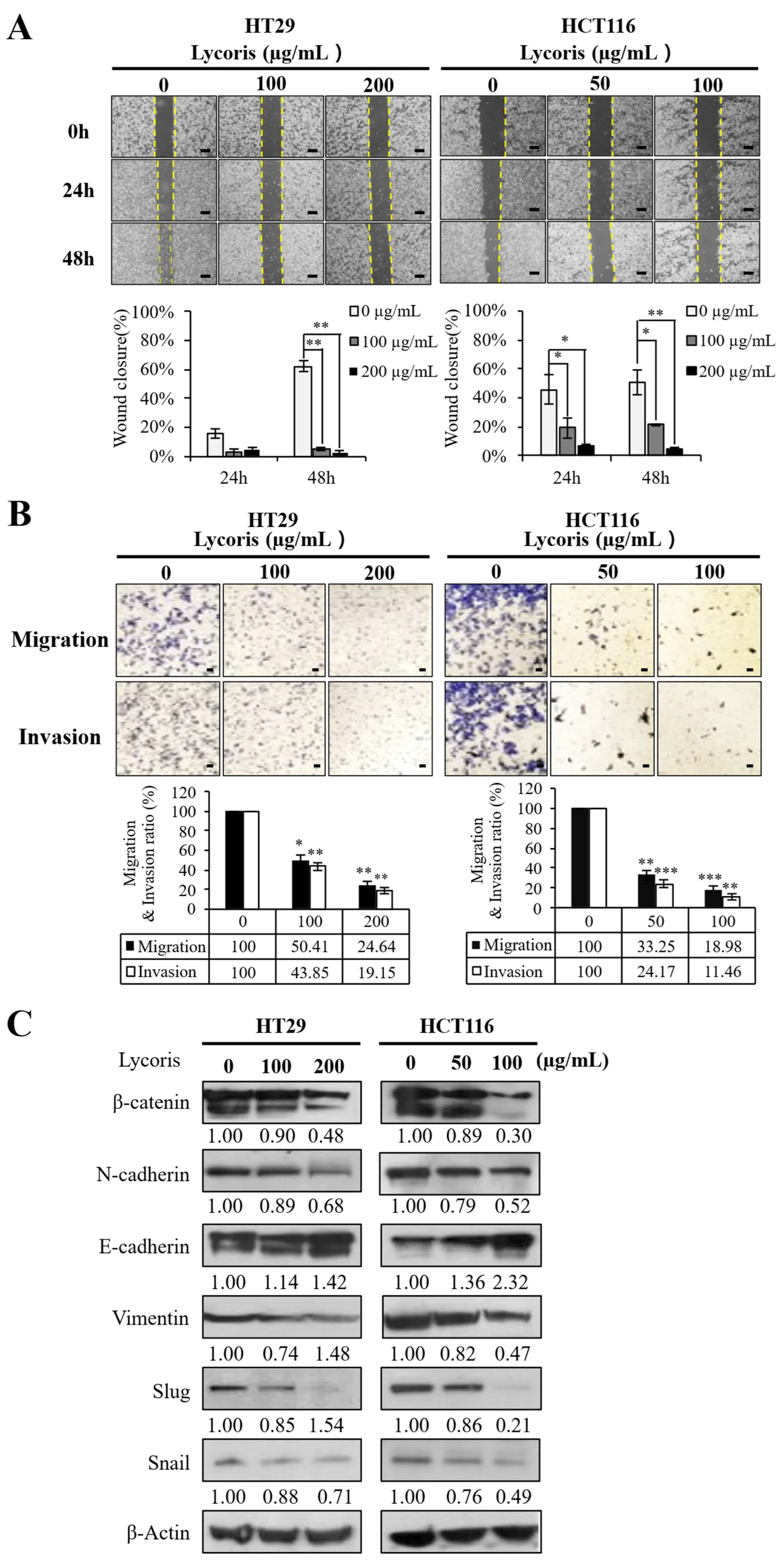
*Lycoris sanguinea* extract suppresses migration and invasion of CRC cells. (**A**) Wound-healing assay of HT29 and HCT116 cells treated with *L. sanguinea* extract for 24 and 48 h. Yellow dashed lines indicate the wound margins used to assess wound closure at each time point. Scale bars, 100 μm. (**B**) Transwell migration and invasion assays. Cells were treated with *L. sanguinea* extract for 48 h, reseeded in Transwell chambers, and migrated or invaded cells were stained with 1% crystal violet. Graphs represent the percentage of migrated or invaded cells. Scale bars, 50 μm. (**C**) Western blot analysis of EMT-related proteins after 48 h treatment with *L. sanguinea* extract or DMSO control. Data are presented as mean ± SD from three independent experiments. Statistical significance: * *p* < 0.05, ** *p* < 0.01, *** *p* < 0.001 versus control.

To assess migration- and invasion-associated phenotypes, wound-healing and Transwell assays were performed in HT29 and HCT116 cells treated with *L. sanguinea* extract. Because these treatment concentrations also reduced cell viability, the migration- and invasion-related results were interpreted together with viability data (Figure 3A,B).

In the wound healing assay, the closure ratio of HT29 cells decreased from 61.92% in controls to 5.26% and 1.98% at 100 and 200 µg/mL, respectively. HCT116 cells showed reductions from 50.92% to 21.51% and 4.63% under the same conditions. To further address the potential contribution of proliferation to wound closure, an additional wound-healing assay was performed under proliferation-controlled conditions using mitomycin C pretreatment. Under this condition, low-dose narciclasine treatment reduced wound closure in both HT29 and HCT116 cells, supporting suppression of migration-associated wound closure under conditions designed to minimize proliferation-dependent effects (Supplementary Figure S1). Consistently, transwell migration of HT29 cells decreased to 50.41 ± 2.11% and 24.64 ± 2.06%, while invasion decreased to 43.85 ± 2.79% and 19.05 ± 1.88% at 100 and 200 µg/mL. In HCT116 cells, migration decreased to 33.25 ± 2.38% and 18.98 ± 2.04%, and invasion to 24.17 ± 2.29% and 11.46 ± 1.72% at the corresponding concentrations.

Western blot analysis of EMT-related proteins revealed downregulation of mesenchymal markers (β-catenin, N-cadherin, vimentin, slug, snail) and upregulation of the epithelial marker E-cadherin (Figure 3C), supporting suppression of EMT.

These results suggest that *L. sanguinea* extract reduces migration- and invasion-associated phenotypes in CRC cells, accompanied by changes in EMT-related protein expression. However, because the treatment concentrations also affected cell viability, these findings should be interpreted cautiously.

### 2.4. Tentative Annotation of Narciclasine as a Major Detected Constituent of Lycoris sanguinea Extract

**Figure 4:**
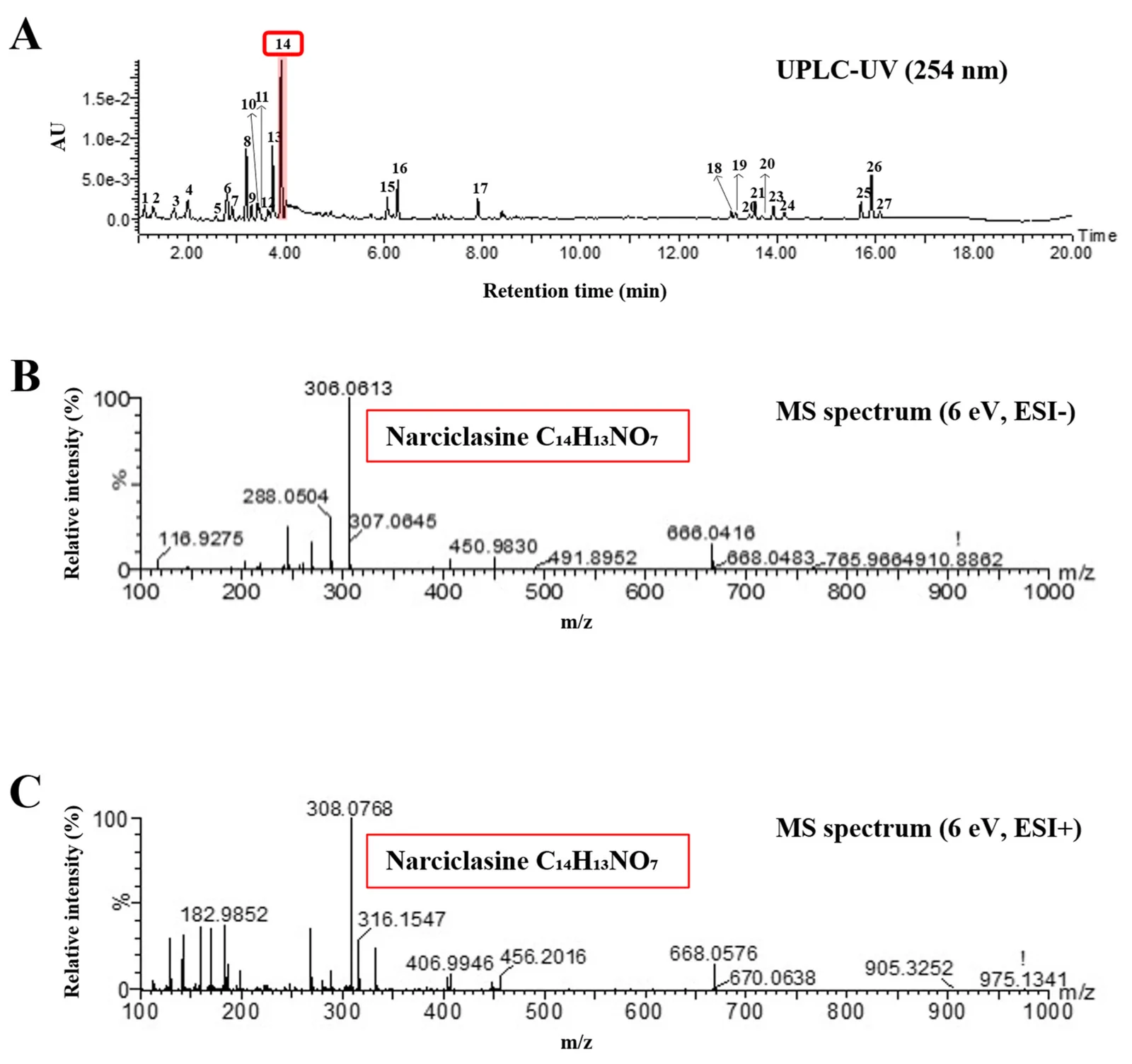
Tentative annotation of narciclasine as a major detected constituent of L. sanguinea extract by UPLC-QTOF/MS analysis. (**A**) UPLC chromatogram at 254 nm showing a major peak at a retention time of 3.90 min. (**B**) MS spectrum in negative ion mode (ESI−) showing a molecular ion peak at *m*/*z* 306.0613. (**C**) MS spectrum in positive ion mode (ESI+) showing a molecular ion peak at *m*/*z* 308.0768. Red boxes highlight peak 14 at a retention time of 3.90 min in panel A and the ten-tative annotation of the corresponding molecular ions as narciclasine (C_14_H_13_NO_7_) in panels B and C. These data support the tentative annotation of the major peak as narciclasine.

To characterize major phytochemical constituents potentially associated with the anticancer effects of *L. sanguinea* extract, UPLC-QTOF/MS analysis was performed. In the UPLC-UV chromatogram at 254 nm, a prominent peak was observed at a retention time of 3.90 min (Figure 4A), which exhibited the highest intensity and was selected for further analysis.

MS analysis revealed a molecular ion peak at *m*/*z* 306.0613 in negative ion mode (ESI^−^) and a corresponding peak at *m*/*z* 308.0768 in positive ion mode (ESI^+^), indicating a neutral mass of 307.0693 (Figure 4B,C). The molecular formula was determined as C_14_H_13_NO_7_, with a calculated monoisotopic mass of 306.0614 and 100% fit confidence, supporting the tentative annotation of the compound as narciclasine. The phytochemical profile of the *Lycoris sanguinea* extract, determined by UPLC-UV, is presented in Table 1.

### 2.5. Integrative Transcriptomic Analyses Prioritize HELLS as a CRC-Associated Candidate Potentially Responsive to Narciclasine

**Figure 5:**
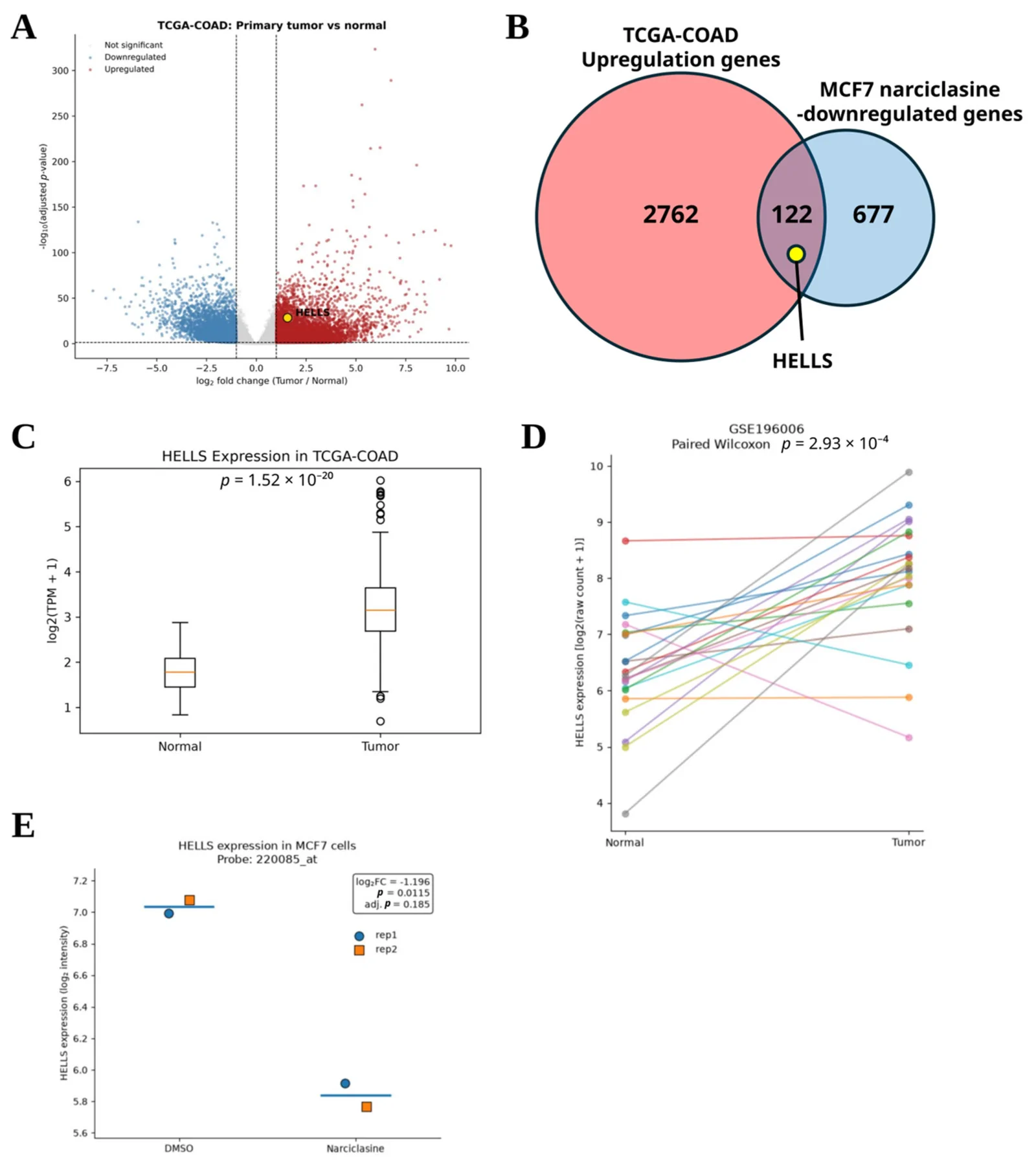
Integrative transcriptomic analyses prioritize HELLS as a CRC-associated candidate potentially responsive to narciclasine. (**A**) Volcano plot showing differentially expressed genes between primary tumor and solid tissue normal samples in the TCGA colon adenocarcinoma (TCGA-COAD) cohort. Both protein-coding and non-coding genes were included in the genome-wide differential expression analysis. *HELLS* is highlighted. Dashed vertical and horizontal lines indicate |log_2_ fold change| = 1 and false discovery rate (FDR) = 0.05, respectively. (**B**) Venn diagram showing the overlap between protein-coding genes significantly upregulated in TCGA-COAD primary tumors relative to solid tissue normal samples and genes showing nominally significant downregulation following narciclasine treatment in MCF7 cells from the GSE85871 dataset. TCGA-COAD genes were selected using log_2_ fold change > 1 and FDR < 0.05, whereas MCF7 genes were selected using log_2_ fold change < −1 and nominal *p* < 0.05. Numbers indicate the genes unique to each dataset and those shared between the two datasets. A total of 122 overlapping genes were identified, including *HELLS*. (**C**) HELLS expression in solid tissue normal (*n* = 41) and primary tumor (*n* = 471) samples from the TCGA-COAD cohort. Expression values are presented as log2 (TPM + 1). Boxes indicate the interquartile range, center lines indicate the median, whiskers extend to 1.5 times the interquartile range, and circles indicate outliers. Statistical significance was evaluated using a two-sided Mann–Whitney U test. (**D**) HELLS expression in 21 patient-matched normal and colorectal tumor tissue pairs from the GSE196006 cohort. Expression values are presented as log2(normalized expression + 1). Lines connect normal and tumor tissues obtained from the same patient. Statistical significance was evaluated using a two-sided paired Wilcoxon signed-rank test. (**E**) HELLS expression in DMSO- and narciclasine-treated MCF7 cells from the GSE85871 dataset (*n* = 2 biological replicates per group). Points represent individual biological replicates, and horizontal lines indicate group means. Differential expression was assessed at the probe level using probe 220085_at. The log2 fold change, nominal *p*-value, and FDR-adjusted *p*-value are indicated.

To identify genes associated with colorectal cancer (CRC) that may also respond to narciclasine, we performed an integrative analysis of publicly available transcriptomic datasets. Differential expression analysis of the TCGA colon adenocarcinoma (TCGA-COAD) cohort revealed extensive transcriptional differences between primary tumors and normal tissues. Among the genes significantly upregulated in tumors, HELLS exhibited a log2 fold change of 1.57 and an FDR-adjusted *p*-value of 3.95 × 10^−29^ (Figure 5A).

We next analyzed the publicly available GSE85871 dataset to identify genes showing decreased expression following narciclasine exposure in MCF7 cells. Protein-coding genes upregulated in TCGA-COAD tumors were compared with genes showing nominally significant downregulation after narciclasine treatment. Using thresholds of log2 fold change > 1 and FDR < 0.05 for the TCGA-COAD dataset, and log2 fold change < −1 and nominal *p* < 0.05 for the MCF7 dataset, 122 overlapping genes were identified. HELLS was included in this intersecting gene set (Figure 5B).

Consistent with the genome-wide differential expression analysis, HELLS expression was significantly higher in TCGA-COAD primary tumor samples than in solid tissue normal samples (normal, *n* = 41; primary tumor, *n* = 471; two-sided Mann–Whitney U test, *p* = 1.52 × 10^−20^; Figure 5C). Based on the DESeq2-derived log2 fold change, HELLS expression was approximately 2.96-fold higher in primary tumors than in normal tissues.

To independently validate the association between HELLS expression and CRC, we examined the GSE196006 cohort, which comprised 21 patient-matched colorectal tumor and normal tissue pairs from patients with early-onset CRC. HELLS expression was significantly elevated in tumor tissues relative to the corresponding normal tissues (two-sided paired Wilcoxon signed-rank test, *p* = 2.93 × 10^−4^; Figure 5D).

In MCF7 cells, narciclasine treatment reduced HELLS expression relative to DMSO treatment, with a log2 fold change of −1.20, corresponding to an approximately 56% reduction in expression (nominal *p* = 0.0115; FDR-adjusted *p* = 0.185; Figure 5E). Although this decrease did not remain statistically significant after multiple-testing correction, this nominal association was used only for exploratory candidate prioritization. Together with the CRC transcriptomic findings, this observation supported the selection of HELLS as a CRC-associated candidate for subsequent functional investigation, rather than as a definitive downstream target of narciclasine. Because GSE85871 was generated from MCF7 breast cancer cells, this dataset was used only as an exploratory pharmacological perturbation resource and not as direct evidence of narciclasine-responsive transcriptional regulation in CRC cells. Therefore, the CRC relevance of HELLS was assessed independently using TCGA-COAD and GSE196006 colorectal cancer datasets, followed by experimental validation in HT29 and HCT116 cells.

### 2.6. siRNA-Mediated HELLS Depletion Sensitizes CRC Cells to L. sanguinea Extract-Associated DNA Damage Signaling and Apoptosis

**Figure 6:**
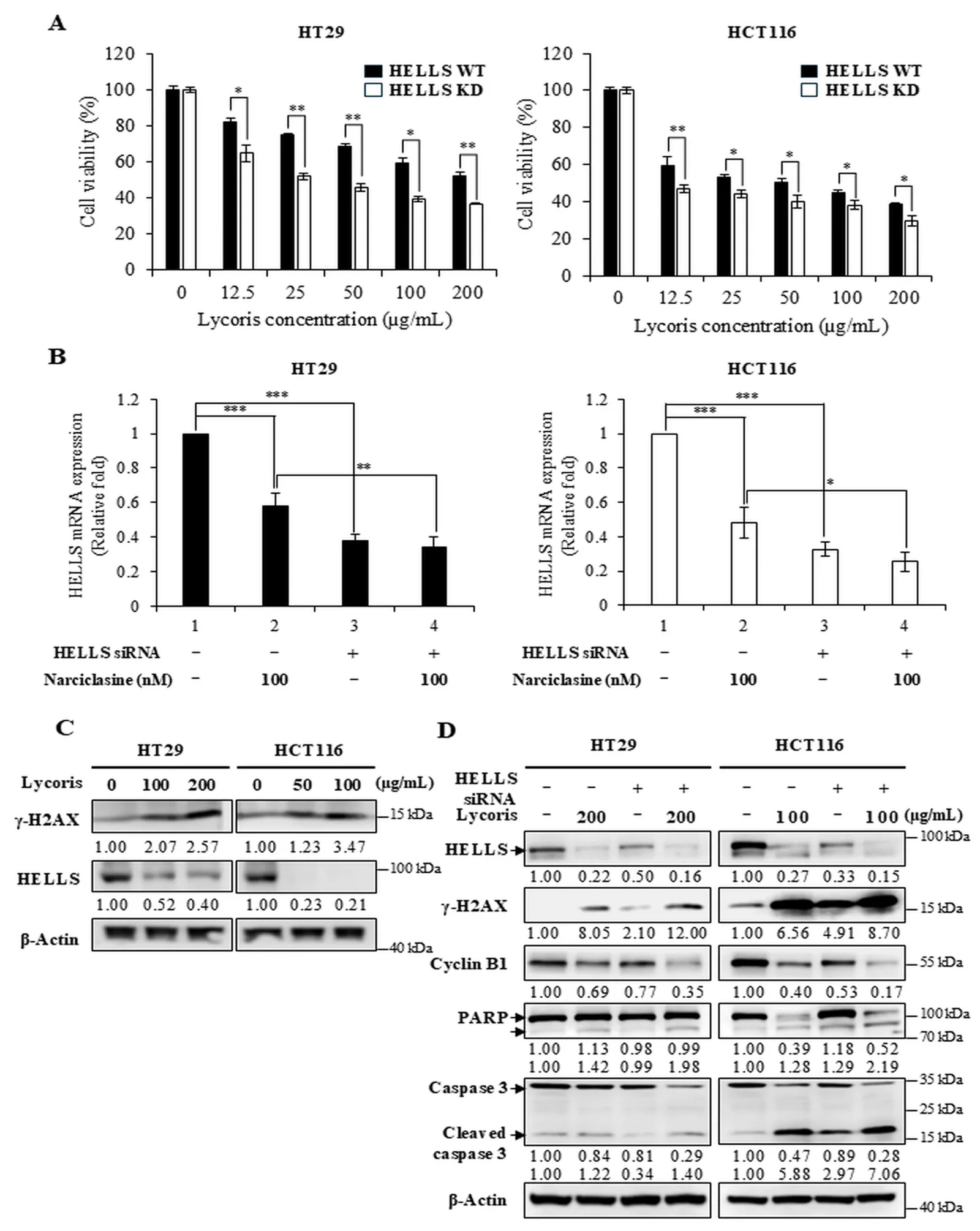
HELLS knockdown enhances *L. sanguinea* extract-associated DNA damage signaling, cell cycle arrest-associated changes, and apoptosis in colorectal cancer cells. (**A**) Cell viability of siControl/siHELLS HT29 and HCT116 cells after 24 h *L. sanguinea* extract treatment. (**B**) RT-qPCR analysis of HELLS mRNA expression in siControl/siHELLS cells following 24 h narciclasine treatment. (**C**) Western blot analysis of HELLS and γ-H2AX protein levels in HT29 and HCT116 cells after 24 h *L. sanguinea* extract treatment. (**D**) Western blot analysis of HELLS, γ-H2AX, cyclin B1, cleaved PARP, and cleaved caspase-3 in siControl/siHELLS cells following *L. sanguinea* extract treatment. Data in (**A**) are presented as mean ± SD from three independent experiments. Statistical significance is indicated as * *p* < 0.05, ** *p* < 0.01. Data in (**B**) are presented as mean ± SD from three independent experiments. Significance was determined by two-way ANOVA with Tukey’s post hoc test (* *p* < 0.05, ** *p* < 0.01, *** *p* < 0.001).

Based on the integrative transcriptomic analysis, HELLS was selected for functional validation. To determine whether HELLS contributes to the anticancer effects of *L. sanguinea* extract, HT29 and HCT116 cells were transfected with non-targeting control siRNA (siControl) or HELLS-targeting siRNA (siHELLS) and subsequently treated with the extract. Cell viability assays showed that siHELLS-transfected cells exhibited increased sensitivity to *L. sanguinea* extract across the tested concentrations (Figure 6A). RT-qPCR analysis confirmed efficient HELLS knockdown and showed further reduction in HELLS mRNA expression following narciclasine treatment, particularly in siHELLS-transfected cells (Figure 6B). Consistently, Western blot analysis demonstrated decreased HELLS protein expression accompanied by increased γ-H2AX accumulation, indicating enhanced DNA damage-associated signaling (Figure 6C).

To further examine whether HELLS depletion potentiates extract-induced cell cycle arrest- and apoptosis-related signaling, siControl- and siHELLS-transfected CRC cells were treated with *L. sanguinea* extract and analyzed by Western blotting. Extract treatment reduced cyclin B1 expression and increased γ-H2AX accumulation, cleaved PARP, and cleaved caspase-3 levels. These effects were more pronounced in siHELLS-transfected cells than in siControl-transfected cells (Figure 6D). These findings suggest that HELLS depletion sensitizes CRC cells to *L. sanguinea* extract-associated DNA damage signaling, G2/M arrest-associated molecular changes, and apoptosis.

### 2.7. Narciclasine Exerts Anticancer Effects Associated with HELLS Downregulation in CRC Cells

**Figure 7:**
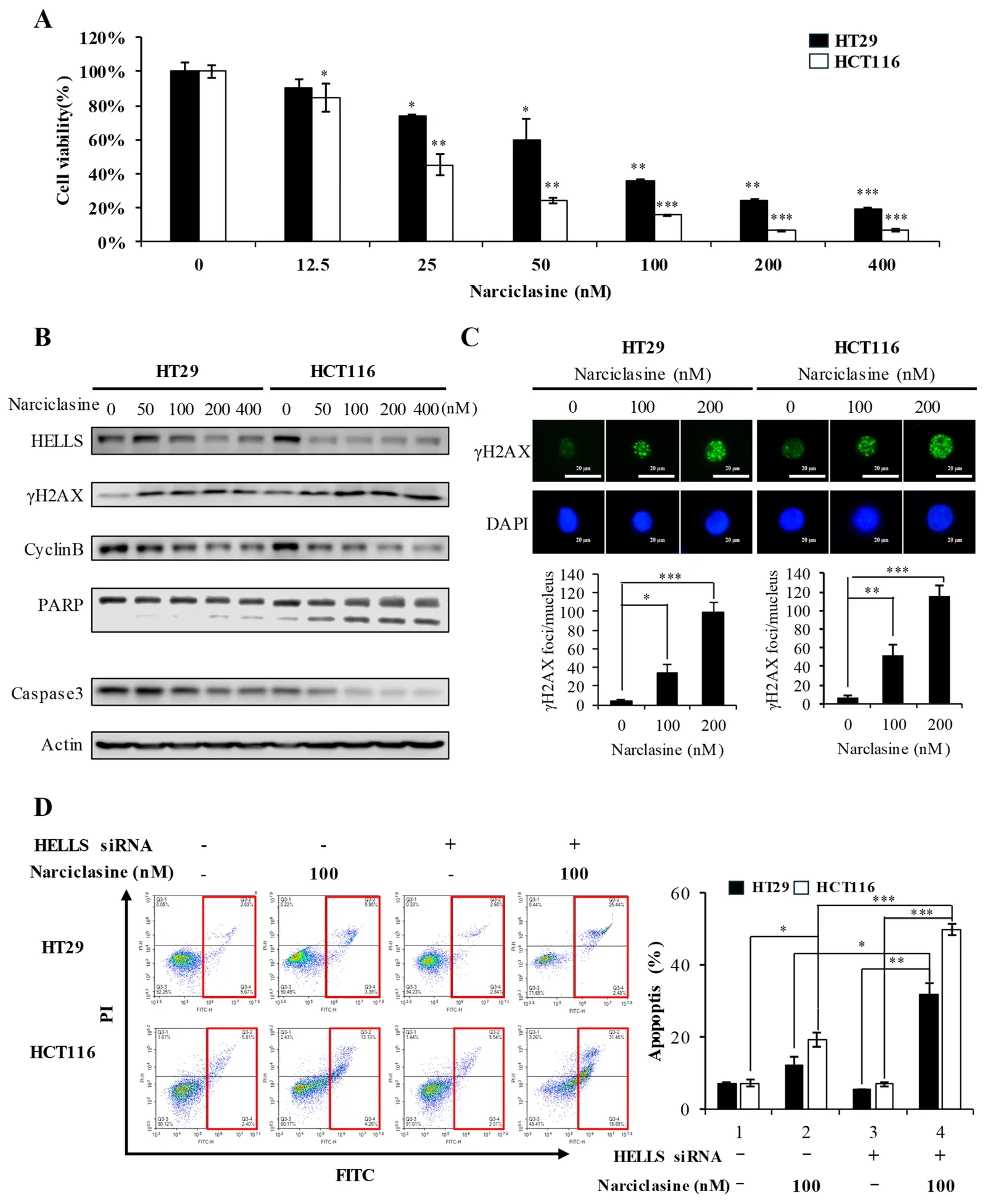

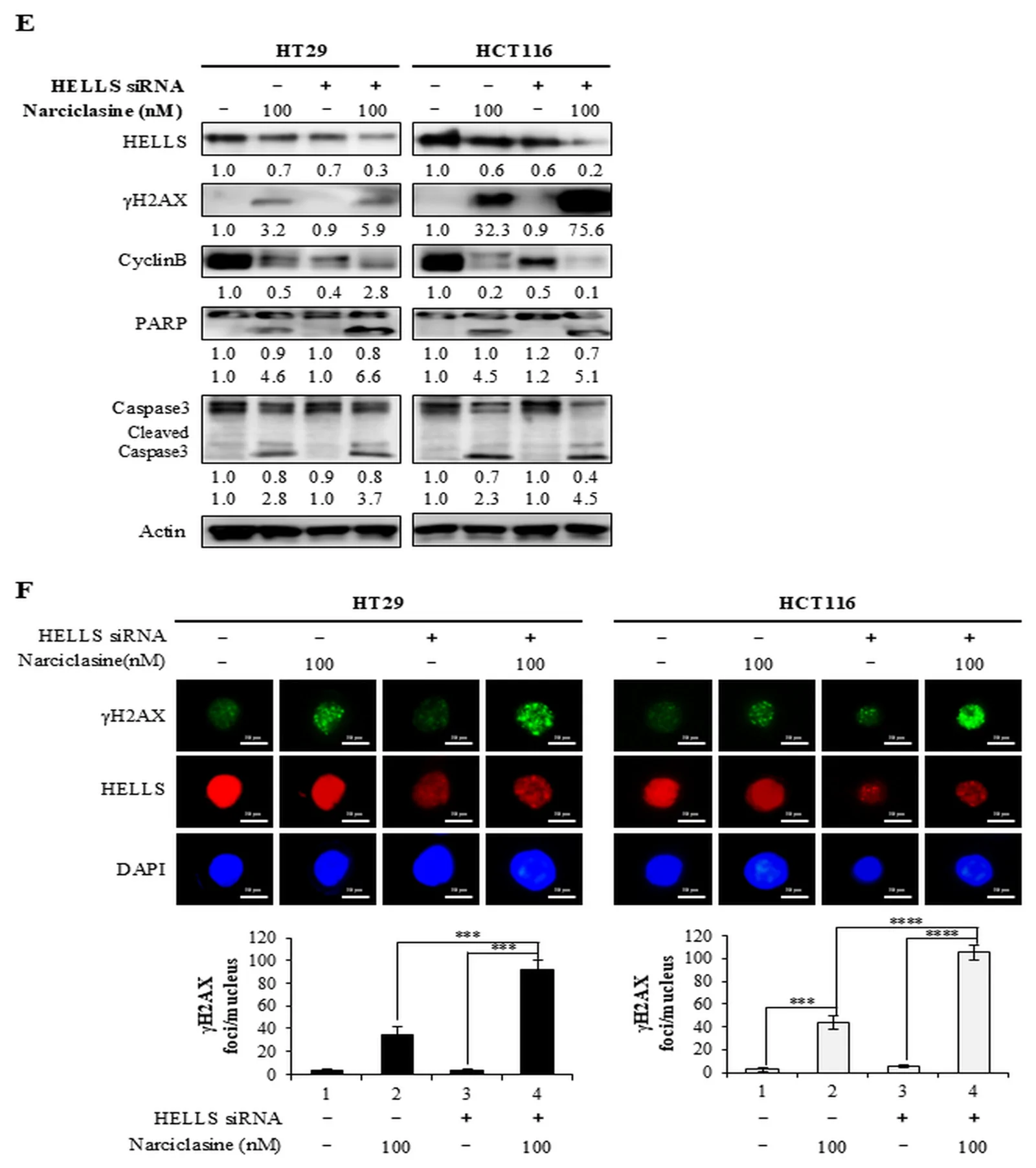
Narciclasine exerts anticancer effects associated with HELLS downregulation in CRC cells. (**A**) Cell viability of HT29 and HCT116 cells treated with narciclasine for 24 h. (**B**) Western blot analysis of HELLS, γ-H2AX, cyclin B1, cleaved PARP, and cleaved caspase-3 following narciclasine treatment. (**C**) Immunofluorescence staining showing nuclear γ-H2AX foci in HT29 and HCT116 cells after narciclasine treatment, supporting activation of DNA damage-associated signaling. (**D**) Apoptosis assays in siControl/siHELLS cells treated with narciclasine, showing increased sensitivity in siHELLS cells. (**E**) Western blot analysis of HELLS, γ-H2AX, cyclin B1, cleaved PARP, and cleaved caspase-3 in siControl/siHELLS cells after treatment. (**F**) Immunofluorescence staining of γ-H2AX foci in siControl/siHELLS cells, showing enhanced DNA damage-associated signaling in siHELLS cells. Data in (**A**,**C**) are presented as mean ± SD from three independent experiments. Statistical significance: * *p* < 0.05, ** *p* < 0.01, *** *p* < 0.001. Data in (**D**,**F**) are presented as mean ± SD from three independent experiments. Statistical significance was determined by two-way ANOVA with Tukey’s post hoc test (* *p* < 0.05, ** *p* < 0.01, *** *p* < 0.001, **** *p* < 0.0001).

To evaluate whether narciclasine alone would recapitulate the anticancer effects of *Lycoris sanguinea* extract, HT29 and HCT116 cells were treated with narciclasine. Treatment significantly reduced cell viability, comparable to the extract (Figure 7A). Western blot analysis showed that narciclasine treatment was associated with reduced HELLS expression and increased γ-H2AX accumulation, suggesting activation of DNA damage-associated signaling (Figure 7B). Immunofluorescence staining confirmed marked accumulation of γ-H2AX nuclear foci in narciclasine-treated cells, supporting activation of DNA damage-associated signaling (Figure 7C). Cyclin B1 levels were decreased, consistent with G_2_/M arrest, and apoptosis was enhanced, as evidenced by PARP cleavage and caspase-3 activation (Figure 7B). To further examine upstream apoptotic events, intracellular ROS-associated fluorescence and mitochondrial cytochrome c release were assessed following narciclasine treatment. Narciclasine increased intracellular ROS-associated CM-H_2_DCFDA fluorescence in both HT29 and HCT116 cells, with a more pronounced increase observed in HCT116 cells. In HCT116 cells, narciclasine reduced cytochrome c levels in the mitochondrial fraction and increased cytochrome c levels in the cytosolic fraction, supporting mitochondrial cytochrome c release during narciclasine-associated apoptosis. In addition, cleaved caspase-9 was detected following narciclasine treatment, further supporting activation of mitochondria-associated apoptotic signaling (Supplementary Figure S2).

**Figure 8:**
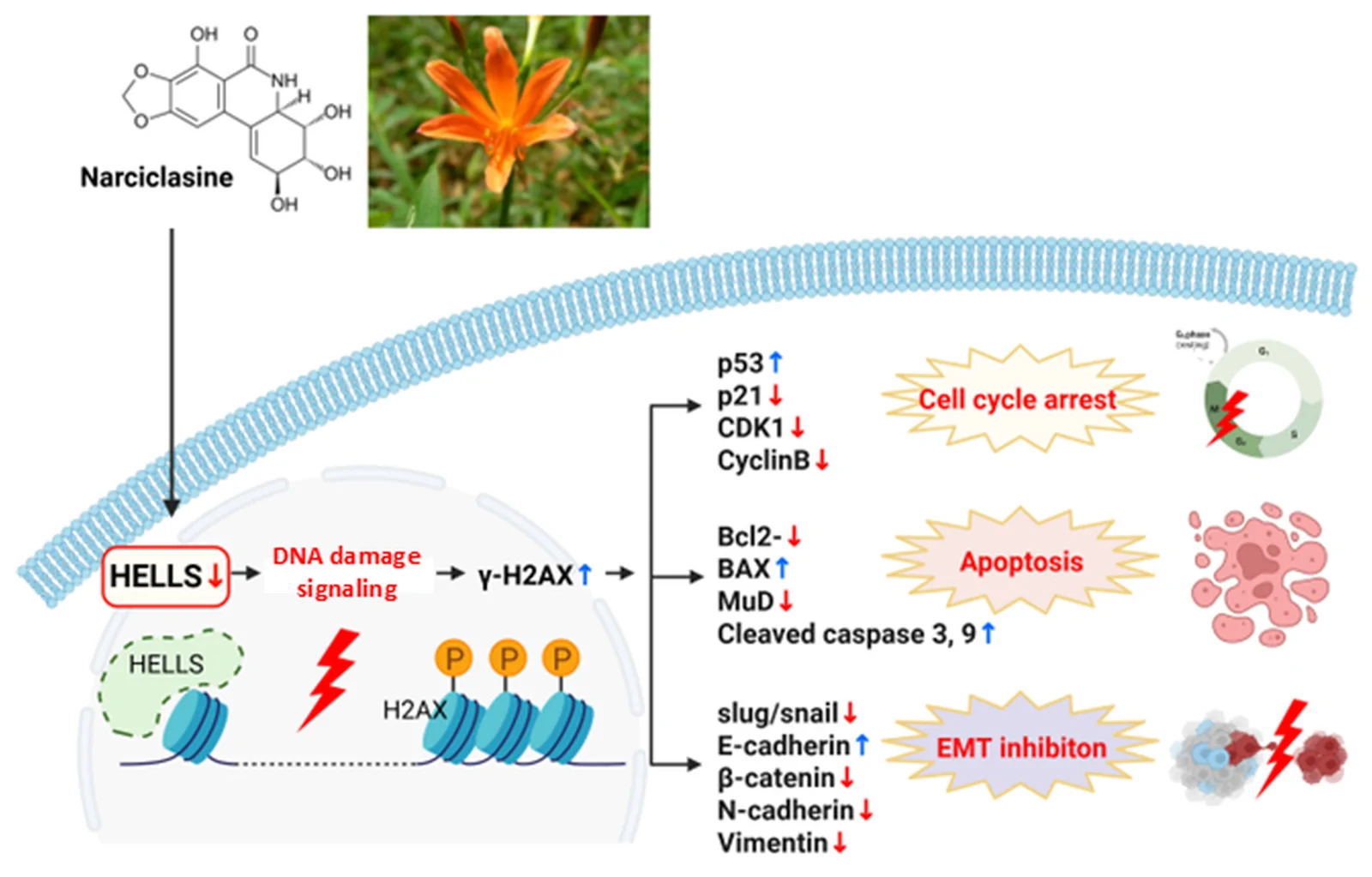
Proposed model of anticancer responses associated with narciclasine treatment and HELLS downregulation in CRC cells. *L. sanguinea* extract suppresses CRC cell viability, clonogenic growth, migration, and invasion while inducing mitochondrial dysfunction, G2/M arrest-associated changes, and apoptosis. UPLC-QTOF/MS analysis tentatively annotated narciclasine as a major detected constituent of the extract. Purified narciclasine recapitulates several anticancer effects of the extract in association with reduced HELLS expression, increased γ-H2AX accumulation, reduced cyclin B1 expression, and enhanced PARP and caspase-3 cleavage. HELLS depletion further sensitizes CRC cells to narciclasine-associated DNA damage signaling and apoptosis, suggesting that HELLS-associated DNA damage tolerance may represent a pharmacological vulnerability in CRC cells. Blue upward arrows indicate increased expression or activation, whereas red downward arrows indicate decreased expression. Black arrows indicate the proposed direction of the associated molecular responses, and other colors are used for visual distinction only.

To examine whether HELLS depletion enhances narciclasine-associated cytotoxic responses, siControl- and siHELLS-transfected CRC cells were treated with narciclasine. Apoptosis assays showed that siHELLS-transfected cells were more sensitive to narciclasine-associated apoptosis than siControl-transfected cells (Figure 7D). Western blot analysis further demonstrated that HELLS depletion enhanced narciclasine-associated γ-H2AX accumulation, cyclin B1 reduction, and PARP and caspase-3 cleavage (Figure 7E). Consistently, γ-H2AX immunofluorescence staining showed more prominent nuclear foci formation in siHELLS-transfected cells following narciclasine treatment (Figure 7F). The proposed model summarizing anticancer responses associated with narciclasine treatment and HELLS downregulation is shown in Figure 8.

Collectively, these results suggest that narciclasine exerts anticancer activity in CRC cells in association with HELLS downregulation, and that HELLS depletion sensitizes CRC cells to narciclasine-associated DNA damage signaling and apoptosis. To further evaluate the selectivity of narciclasine toward CRC cells, we compared its cytotoxic effect in HIEC-6 non-malignant intestinal epithelial cells and CRC cells. Basal HELLS expression was lower in HIEC-6 cells than in HT29 and HCT116 cells. Consistently, HIEC-6 cells were less sensitive to narciclasine than CRC cells under the same treatment conditions. At 100 nM narciclasine, HIEC-6 cell viability remained at 82.7%, whereas HT29 and HCT116 cell viability decreased to 40.0% and 17.7%, respectively (Supplementary Figure S3). These results suggest that narciclasine shows preferential cytotoxicity toward CRC cells compared with non-malignant intestinal epithelial cells under the present in vitro conditions.

## 3. Discussion

This study demonstrates that *Lycoris sanguinea* extract and narciclasine, which was tentatively annotated as a major detected constituent of the extract, suppress malignant phenotypes of CRC cells in association with HELLS downregulation. Both the extract and narciclasine reduced cell viability, inhibited migration and invasion, induced G_2_/M arrest, and promoted apoptosis. At the molecular level, these effects were accompanied by HELLS downregulation, DNA damage-associated signaling, checkpoint-associated molecular changes, apoptotic activation, and changes in EMT-related protein expression.

The integrative transcriptomic analysis provided an additional rationale for focusing on HELLS as a candidate mediator of narciclasine responsiveness. HELLS was significantly upregulated in TCGA-COAD tumors and independently validated in patient-matched colorectal tumor tissues from the GSE196006 cohort, supporting its relevance to CRC biology. Although the narciclasine perturbation dataset was derived from MCF7 cells and the decrease in HELLS expression did not remain significant after multiple-testing correction, the direction and magnitude of HELLS downregulation supported its prioritization as a candidate gene potentially responsive to narciclasine exposure. Therefore, the subsequent siRNA-based functional validation in CRC cells strengthened the link between HELLS depletion and increased sensitivity to narciclasine-associated DNA damage and apoptosis. Because HELLS downregulation in GSE85871 did not remain significant after FDR correction, we do not interpret HELLS as a principal downstream target of narciclasine based on this dataset. Rather, HELLS was selected as a functionally relevant candidate, and its relevance was subsequently examined in CRC cells. Thus, the MCF7-derived dataset was used only for exploratory candidate prioritization, whereas the biological relevance of HELLS in CRC was supported by CRC transcriptomic datasets and experimental validation in HT29 and HCT116 cells.

HELLS (also LSH or SMARCA6) is a sucrose non-fermenting 2 (SNF2)-family ATP-dependent chromatin remodeler with essential roles in genome maintenance and oncogenesis [20,21,22,26,27,28]. By facilitating chromatin accessibility at replication origins, HELLS ensures replication fork stability; its depletion induces fork stalling and collapse, leading to replication stress and γ-H2AX accumulation [22,23,25]. Previous studies have shown that HELLS can contribute to homologous recombination repair through BRCA1- and RAD51-associated mechanisms and that HELLS loss may impair double-strand break repair [24]. In the present study, narciclasine treatment was associated with reduced HELLS expression and increased γ-H2AX accumulation, suggesting enhanced DNA damage-associated signaling. However, activation or impairment of specific DNA repair pathways, including ATM/ATR–CHK1/CHK2 or BRCA1/RAD51 signaling, was not directly examined [29,30]. Although γ-H2AX accumulation is consistent with DNA damage-associated signaling, it does not by itself establish direct induction of DNA double-strand breaks, because γ-H2AX can also arise secondary to replication stress or apoptosis.

HELLS downregulation may be linked to checkpoint-associated responses that restrict mitotic progression in the presence of DNA damage; however, activation of specific ATR–CHK1 or ATM–CHK2 signaling components was not directly assessed in this study. Therefore, the observed reduction in cyclin B1 and increase in PARP and caspase-3 cleavage should be interpreted as G2/M checkpoint- and apoptosis-associated molecular changes rather than direct evidence of a defined DNA repair pathway. Together, these findings support an association among narciclasine treatment, HELLS downregulation, γ-H2AX accumulation, G2/M arrest-associated changes, and apoptosis, while the precise DNA repair or checkpoint pathways involved remain to be determined. Although the present findings were obtained in colorectal cancer cells, HELLS itself is not a colorectal cancer-specific factor. HELLS has been implicated in chromatin remodeling, genome maintenance, DNA damage tolerance, and malignant progression across multiple cancer types [27,28,31]. Therefore, the observed association between narciclasine treatment, HELLS downregulation, and DNA damage-associated signaling may represent a broader cancer-cell vulnerability rather than a CRC-restricted mechanism. However, because the present experimental validation was limited to HT29 and HCT116 CRC cells, further studies using additional CRC models, other cancer types, and non-malignant epithelial cells will be required to define the cancer-type specificity and therapeutic selectivity of this response.

Beyond genome integrity, HELLS contributes to transcriptional programs associated with EMT. By remodeling chromatin, HELLS likely enhances accessibility at promoters of EMT-inducing transcription factors such as snail, slug, and ZEB1, promoting mesenchymal gene expression [32,33]. In our study, treatment with L. sanguinea extract, in which narciclasine was tentatively annotated as a major detected constituent, decreased mesenchymal markers (N-cadherin, vimentin, slug) and increased E-cadherin expression, suggesting that HELLS downregulation may contribute to EMT-associated changes and reduced metastatic potential.

However, because the concentrations used in the original migration and invasion assays also reduced cell viability, the observed suppression of motility may partially reflect reduced proliferation or survival, in addition to EMT modulation. To partially address this issue, an additional wound-healing assay using mitomycin C pretreatment showed that low-dose narciclasine reduced wound closure under proliferation-controlled conditions. Nevertheless, because the Transwell migration and invasion assays were not repeated under proliferation-controlled conditions, these data should be interpreted cautiously. Additional Transwell assays using sub-cytotoxic concentrations or mitomycin C pretreatment will be required to further clarify migration- and invasion-specific effects of narciclasine or *L. sanguinea* extract.

Importantly, HCT116 cells were more sensitive to narciclasine than HT29 cells, which may be related, at least in part, to differences in basal HELLS expression or other cell-line-specific molecular features. These observations suggest that HELLS-associated chromatin remodeling and DNA damage tolerance may influence cellular sensitivity to narciclasine, although additional CRC models will be required to validate HELLS as a predictive biomarker. Clinically, HELLS overexpression has been associated with advanced CRC and poor prognosis, suggesting that HELLS may warrant further investigation as a candidate biomarker related to narciclasine responsiveness.

Natural products remain invaluable sources of anticancer agents, exemplified by frontline drugs such as paclitaxel and irinotecan [34,35,36,37]. While narciclasine is known as a broadly cytotoxic Amaryllidaceae alkaloid [14,38,39], our findings suggest that its anticancer effects in CRC are associated with HELLS downregulation, affecting DNA replication, repair, apoptosis, and EMT-related phenotypes. Although narciclasine was tentatively annotated as a major detected constituent of *L. sanguinea* extract, the extract contained multiple additional peaks in the UPLC-UV profile. Therefore, the biological effects of the crude extract cannot be attributed exclusively to narciclasine. Other constituents may also contribute to the observed anticancer activity, and additive or synergistic interactions among extract components cannot be excluded. Further activity-guided fractionation, isolation of additional constituents, and quantitative phytochemical profiling will be required to define the relative contribution of narciclasine and other extract components.

From a translational perspective, narciclasine should be considered a natural-product-derived lead compound or pharmacological tool rather than an immediately applicable therapeutic agent. Although narciclasine has shown potent anticancer activity in several preclinical models, its further development will require careful evaluation of pharmacokinetic properties, bioavailability, metabolic stability, tissue distribution, formulation and delivery strategies, systemic toxicity, and therapeutic index. In particular, because narciclasine exhibits broad cytotoxic activity, defining tumor-selective vulnerabilities and optimizing delivery approaches will be essential before considering clinical translation. Therefore, the present findings should be interpreted as supporting further preclinical investigation of narciclasine-associated HELLS vulnerabilities, rather than establishing narciclasine as a clinically ready CRC therapeutic candidate.

Several limitations should be considered. First, this study was performed using two CRC cell lines, HT29 and HCT116, and therefore may not fully represent the molecular heterogeneity of colorectal cancer. Although consistent responses were observed in both models, further validation in additional CRC cell lines with distinct molecular characteristics, as well as in three-dimensional spheroid models, patient-derived organoids, and in vivo CRC models, will be required to establish the broader applicability and translational relevance of the observed responses before narciclasine can be considered a therapeutic candidate for CRC. Second, although UPLC-QTOF/MS analysis tentatively annotated narciclasine as a major detected constituent of *L. sanguinea* extract, definitive structural confirmation using an authentic standard, MS/MS fragmentation analysis, and/or NMR analysis would further strengthen the phytochemical characterization. In addition, because an authentic-standard-based calibration curve was not used, the absolute content of narciclasine in the extract could not be determined. Therefore, the relative peak area observed in the UPLC-UV profile should not be interpreted as quantitative abundance or purity. Future studies using quantitative LC-MS/MS or HPLC analysis with an authentic narciclasine standard will be required to determine extraction yield, purity, and absolute narciclasine content. In addition, because the UPLC-UV profile showed multiple additional peaks, the contribution of other constituents and possible additive or synergistic interactions cannot be excluded. Further activity-guided fractionation and quantitative phytochemical profiling will be required to define the relative contribution of narciclasine and other extract components. Third, although the additional HIEC-6 cell viability assay provided an initial assessment of narciclasine selectivity toward CRC cells, only one non-malignant intestinal epithelial cell line was examined. Therefore, further studies using additional non-malignant colon/intestinal epithelial models and in vivo toxicity analyses will be required to define the therapeutic window more rigorously. Fourth, although additional experiments showed increased ROS-associated CM-H_2_DCFDA fluorescence, mitochondrial cytochrome c release, and caspase-9 cleavage following narciclasine treatment, the upstream apoptotic signaling events were not fully defined. Future studies assessing specific oxidative stress-associated markers and ROS scavenger-based rescue experiments will be required to define the apoptotic pathway more precisely. Fifth, functional activation or impairment of specific DNA damage repair and checkpoint pathways, including ATM/ATR–CHK1/CHK2 and BRCA1/RAD51 signaling, was not directly examined. Future studies using phosphorylated checkpoint kinase analysis, BRCA1/RAD51 recruitment assays, and DNA repair reporter assays will be required to define the specific DNA damage repair pathways involved. Sixth, although γ-H2AX accumulation suggests activation of DNA damage-associated signaling, γ-H2AX alone does not establish direct DNA double-strand break formation. Future studies using comet assays, 53BP1 foci analysis, or other DNA damage assays will be required to determine whether narciclasine directly induces DNA double-strand breaks or whether γ-H2AX accumulation occurs secondary to replication stress and/or apoptosis. Finally, although siRNA-mediated HELLS knockdown showed that HELLS depletion sensitized CRC cells to narciclasine-associated DNA damage signaling and apoptosis, these loss-of-function experiments do not establish whether HELLS downregulation is required for narciclasine-induced anticancer activity. HELLS overexpression or rescue experiments will be required in future studies to determine whether restoration of HELLS expression can attenuate narciclasine-induced γ-H2AX accumulation, G2/M arrest-associated signaling, and apoptosis.

## 4. Materials and Methods

### 4.1. Preparation of Lycoris sanguinea Extract

The methanolic extract of *Lycoris sanguinea* Maxim. (KPM007-035) was obtained from the Korea Plant Extract Bank, Korea Research Institute of Bioscience and Biotechnology, Ochang, Republic of Korea. The extract was prepared from bulbs using methanol extraction according to the supplier’s standardized protocol.

The methanolic extract of *Lycoris sanguinea* was dissolved in dimethyl sulfoxide (DMSO) to prepare a stock solution of 200 mg/mL and stored at −20 °C until use. The stock solution was diluted in culture medium immediately before treatment, and the final DMSO concentration was maintained at ≤0.1% in all experimental groups, including vehicle controls.

### 4.2. Narciclasine Preparation and Treatment

Narciclasine was purchased from MedChemExpress (HY-16563) (Monmouth Junction, NJ, USA) with a purity of ≥98%. Narciclasine was dissolved in DMSO to prepare a 10 mM stock solution and stored at −20 °C. For cell-based experiments, the stock solution was diluted in culture medium immediately before treatment to the indicated final concentrations. The final concentration of DMSO was kept constant at ≤0.1% in all treatment and vehicle control groups. Cells were treated with narciclasine for 24 h unless otherwise indicated.

### 4.3. Cell Culture

Human CRC cell lines HT29 and HCT116 were obtained from the American Type Culture Collection (ATCC, Manassas, VA, USA). Cells were cultured in McCoy’s 5A medium supplemented with 10% fetal bovine serum and 1% penicillin–streptomycin at 37 °C in a humidified atmosphere containing 5% CO_2_. HIEC-6 non-malignant human intestinal epithelial cells were kindly provided by Prof. Soo-Jong Hong at Asan Medical Center and used to assess the selectivity of narciclasine toward CRC cells. HIEC-6 cells were cultured in Opti-MEM supplemented with 20 mM HEPES, 10 mM GlutaMAX, 10 ng/mL epidermal growth factor, 4% fetal bovine serum, and 1% penicillin–streptomycin at 37 °C in a humidified atmosphere containing 5% CO_2_. Cells were used before passage 20. Cells were routinely tested and confirmed to be free of mycoplasma contamination using a detection kit (Koma Biotech, Seoul, Republic of Korea).

### 4.4. Cell Viability Assay

Cell viability was assessed using a Cell Counting Kit-8 assay (CCK-8; Dojindo, Kumamoto, Japan). Cells were seeded in 96-well plates at 1 × 10^4^ cells/well and allowed to attach overnight. Cells were then treated with *L. sanguinea* extract or narciclasine at the indicated concentrations for 24 h. After treatment, CCK-8 reagent was added to each well and incubated for 1 h at 37 °C. Absorbance was measured at 450 nm using a SpectraMax M2 microplate reader. (Molecular Devices, San Jose, CA, USA) Cell viability was calculated as a percentage of the vehicle-treated control.

### 4.5. Apoptosis Assay

Apoptosis was analyzed using an eBioscience™ Annexin V Apoptosis Detection Kit (Thermo Fisher Scientific, Waltham, MA, USA) according to the manufacturer’s instructions. After treatment, cells were collected, washed with cold DPBS, resuspended in 1× binding buffer, and stained with FITC Annexin V and propidium iodide for 15 min at room temperature in the dark. Apoptotic cells were quantified using a NovoCyte 1000 flow cytometer (ACEA Biosciences, San Diego, CA, USA). Early and late apoptotic populations were defined as Annexin V^+^/PI^−^ and Annexin V^+^/PI^+^ cells, respectively.

### 4.6. JC-1 Staining Assay

Mitochondrial membrane potential was measured using JC-1 staining. After treatment, cells were incubated with 2 µM JC-1 at 37 °C for 30 min, washed with DPBS, and analyzed by flow cytometry. Loss of mitochondrial membrane potential was determined by an increase in JC-1 green fluorescence and/or a decrease in the red/green fluorescence ratio.

### 4.7. Measurement of Intracellular ROS

Intracellular ROS production was assessed using CM-H_2_DCFDA staining. HT29 cells were treated with narciclasine at 100 or 200 nM for 24 h, whereas HCT116 cells were treated with narciclasine at 50 or 100 nM for 24 h. After treatment, cells were incubated with 20 μM CM-H_2_DCFDA (Invitrogen, Thermo Fisher Scientific, Eugene, OR, USA; Cat. No. C6827) for 1 h at 37 °C. Cellular fluorescence was measured using a NovoCyte Flow Cytometer System (Agilent Technologies, Santa Clara, CA, USA), and CM-H_2_DCFDA fluorescence intensity was quantified to determine intracellular ROS-associated fluorescence relative to untreated controls.

### 4.8. Cell Cycle Assay

Cell cycle distribution was analyzed using flow cytometry after PI staining. Briefly, treated cells were fixed in 70% ethanol at −20 °C, incubated with RNase A, and stained with PI. DNA content was analyzed using a NovoCyte 1000 flow cytometer, and the percentages of cells in G0/G1, S, and G2/M phases were quantified using NovoExpress. (version 1.6.2).

### 4.9. Clonogenic Assay

Cells (2.5 × 10^5^ cells/well) were seeded in 6-well plates, treated with *Lycoris* for 24 h, and then reseeded at 500 cells/well in 6-well plates. Colonies were cultured for 10 days, fixed with 4% paraformaldehyde, permeabilized with 100% methanol, and stained with 1% crystal violet. Colony numbers were quantified using ImageJ software (v1.8.0, NIH, Bethesda, MD, USA).

### 4.10. Wound Healing Assay

Wound healing assays were performed to evaluate the effect of *L. sanguinea* extract on cell motility. Because the extract also affected cell viability, migration-related results were interpreted together with viability data. Images were acquired at 0 and 24/48 h after scratching, and wound closure was quantified using ImageJ. For the additional proliferation-controlled wound-healing assay, cells were pretreated with mitomycin C at 5 μg/mL for 2 h to minimize the contribution of cell proliferation. Cells were then treated with narciclasine at 12.5 nM, and wound images were captured at 0 and 48 h. Wound closure was quantified as [(wound area at 0 h − wound area at 48 h)/wound area at 0 h] × 100.

### 4.11. Transwell Migration and Invasion Assay

For migration assays, treated cells were seeded in the upper chamber of 8 µm pore-size Transwell inserts in serum-reduced medium, whereas the lower chamber contained medium supplemented with 10% FBS as a chemoattractant. For invasion assays, inserts were coated with Matrigel before cell seeding. After incubation, migrated or invaded cells on the lower membrane surface were fixed, stained with crystal violet, imaged, and counted in five randomly selected fields per insert.

### 4.12. Mitochondrial and Cytosolic Fractionation

Mitochondrial and cytosolic fractions were prepared to assess cytochrome c release. Cells were harvested by centrifugation and washed with ice-cold NKM buffer containing 1 mM Tris-HCl, pH 7.4, 130 mM NaCl, 5 mM KCl, and 7.5 mM MgCl_2_. Cell pellets were resuspended in homogenization buffer containing 10 mM Tris-HCl, pH 6.7, 10 mM KCl, 0.15 mM MgCl_2_, 1 mM PMSF, and 1 mM DTT and incubated on ice for 10 min. Cells were then disrupted using a Dounce homogenizer, and the homogenates were gently mixed with 2 M sucrose solution. Unbroken cells, nuclei, and large cellular debris were removed by centrifugation at 1200× *g* for 5 min. The resulting supernatants were centrifuged again under the same conditions and subsequently centrifuged at 7000× *g* for 10 min to obtain crude mitochondrial pellets. The post-mitochondrial supernatants were collected as cytosol-enriched fractions and used for immunoblot analysis. Crude mitochondrial pellets were resuspended in mitochondrial suspension buffer containing 10 mM Tris-HCl, pH 6.7, 0.15 mM MgCl_2_, 0.25 M sucrose, 1 mM PMSF, and 1 mM DTT and centrifuged at 9500× *g* for 5 min. The mitochondria-enriched pellets were lysed in RIPA buffer and centrifuged at 10,000× *g* for 5 min. Supernatants containing solubilized mitochondrial proteins were collected for immunoblot analysis. HSP60 and β-actin were used as mitochondrial and cytosolic fraction markers, respectively.

### 4.13. Western Blot Analysis

Cells (1 × 10^6^) were treated with *L. sanguinea* extract for 24 h, lysed in PRO-PREP™ (iNtRON Biotechnology, Seoul, Republic of Korea) containing protease inhibitors, and protein concentrations were determined using a DC protein assay (Bio-Rad, Hercules, CA, USA). Lysates (30 µg) were resolved by SDS-PAGE and transferred to Polyvinylidene difluoride (PVDF) membranes (Bio-Rad). Membranes were blocked with 5% skim milk and incubated overnight at 4 °C with primary antibodies, followed by HRP-conjugated secondary antibodies. Primary antibodies included HELLS, γ-H2AX, p53, p21, cyclin B1, CDK1, cyclin A2, CDK2, PARP, MuD, bcl-2, bad, bax, caspase-3, cleaved caspase-3, β-catenin, N-cadherin, E-cadherin, vimentin, slug, snail, cytochrome c, HSP60, and caspase-9 (Supplementary Table S1). An in-house-developed monoclonal antibody against MuD was used for immunoblotting. Protein bands were visualized using enhanced chemiluminescence and detected using X-ray film. Band intensities were quantified using ImageJ software and normalized to β-actin or GAPDH. For quantitative analyses, densitometric values were calculated from at least three independent biological experiments.

### 4.14. qRT-PCR

Total RNA was extracted using TRIzol reagent (Invitrogen, Thermo Fisher Scientific, Carlsbad, CA, USA) according to the manufacturer’s instructions. RNA concentration and purity were measured using NanoDrop One spectrophotometer (Thermo Fisher Scientific, Wilmington, DE, USA). cDNA was synthesized from 1 μg of total RNA using AccuPower RT Premix (Bioneer, Daejeon, Republic of Korea). qPCR was performed using SYBR qPCR Mix on a CFX96 Real-Time PCR System (Bio-Rad Laboratories, Hercules, CA, USA). Relative mRNA expression was calculated using the 2^−ΔΔCt^ method with GAPDH as the internal control. Primer sequences are listed in Supplementary Table S2.

### 4.15. siHELLS Knockdown

For transient HELLS knockdown, cells were transfected with HELLS-targeting siRNA or non-targeting control siRNA using Lipofectamine 2000 (Invitrogen, Thermo Fisher Scientific, Carlsbad, CA, USA) according to the manufacturer’s instructions. Briefly, cells were seeded to reach 50% confluency at the time of transfection and transfected with 100 nM siRNA in Opti-MEM medium. After 6 h, the medium was replaced with complete medium, and cells were incubated for an additional 18 h before treatment. Knockdown efficiency was confirmed by qRT-PCR and Western blotting. The siRNA sequences were as follows: siHELLS, sense 5′-GUUGUUUAUCGCCUUGUUA-3′ and antisense 5′-UAACAAGGCGAUAAACAAC-3′ and siControl, sense 5′-UUCUCCGAACGUGUCACGUTT-3′ and antisense 5′-AAACGUGACACGUUCGGAGAA-3′.

### 4.16. UPLC-QTOF/MS Analysis

UPLC-QTOF/MS analysis was performed using a Waters ACQUITY UPLC system coupled to a XEVO-G2 XS QTOF mass spectrometer (Waters Corporation, Milford, MA, USA). Separation was achieved on an ACQUITY UPLC BEH C18 column (Waters, Milford, MA, USA) using 0.1% formic acid in water as solvent A and acetonitrile as solvent B. The gradient program was as follows: 0–1 min, 2% B; 1–10 min, 2–30% B; 10–15 min, 30–95% B; 15–18 min, 95% B; 18–19 min, 95–2% B; 19–23 min, 2% B. The injection volume was 3 μL, column temperature was maintained at 35 °C, and the flow rate was 0.4 mL/min. MS data were acquired in both positive and negative electrospray ionization modes over an *m*/*z* range of 100–1500. The major peak was tentatively annotated as narciclasine based on accurate mass, isotope pattern, UV absorbance, and comparison with published spectral data and database information.

### 4.17. Immunofluorescence Staining

Cells grown on glass coverslips were fixed with 4% paraformaldehyde, permeabilized with 0.1% Triton X-100, and blocked with 1% horse serum. Cells were incubated with anti-γ-H2AX antibody (1:1000) overnight at 4 °C, followed by FITC-conjugated secondary antibody (1:500). Nuclei were counterstained with DAPI. Images were acquired using an IX73 fluorescence microscope (Evident Scientific, Hachioji-shi, Japan) under identical exposure settings. γ-H2AX foci were quantified in at least 40 nuclei per group using ImageJ.

### 4.18. Bioinformatics Analysis of Public Transcriptomic Datasets

RNA-sequencing data from The Cancer Genome Atlas colon adenocarcinoma (TCGA-COAD) cohort were obtained from the National Cancer Institute Genomic Data Commons (GDC) Data Portal. The dataset included 471 primary tumor samples and 41 solid tissue normal samples. Differential expression analysis between primary tumor and normal tissues was performed using PyDESeq2 (version 0.5.4). Genes with a log2 fold change > 1 and a false discovery rate (FDR)-adjusted *p* value < 0.05 were considered significantly upregulated in CRC tumors. For visualization of HELLS expression, transcript abundance values were presented as log2(TPM + 1), and differences between primary tumor and normal tissues were assessed using a two-sided Mann–Whitney U test.

To explore genes potentially responsive to narciclasine exposure, the publicly available GSE85871 dataset was obtained from the Gene Expression Omnibus (GEO). Because publicly available narciclasine-treated CRC transcriptomic datasets were not available, this dataset, generated from narciclasine-treated MCF7 cells, was used as an exploratory pharmacological perturbation dataset. Probe-level expression values were compared between narciclasine- and DMSO-treated samples using a two-sided Welch’s t-test, followed by Benjamini–Hochberg correction for multiple testing. For exploratory candidate screening, genes with a log2 fold change < −1 and a nominal *p* value < 0.05 were considered nominally downregulated after narciclasine treatment.

To independently validate HELLS expression in CRC tissues, the GSE196006 dataset was obtained from GEO. This cohort included 21 patient-matched colorectal tumor and adjacent normal tissue pairs from patients with early-onset CRC. HELLS expression was visualized as log2(normalized expression + 1), and differences between matched tumor and normal tissues were assessed using a two-sided paired Wilcoxon signed-rank test.

Protein-coding genes significantly upregulated in TCGA-COAD tumors were intersected with genes nominally downregulated after narciclasine treatment in GSE85871. Duplicate gene symbols were removed before comparison. The overlapping gene set was used to prioritize candidate genes associated with both CRC progression and potential responsiveness to narciclasine exposure. All data processing, statistical analyses, and visualization were performed using Python (version 3.10.12).

### 4.19. Statistical Analysis

Statistical analyses were performed using GraphPad Prism (version 8). Data are presented as mean ± standard deviation (SD) from at least three independent biological experiments. Before applying parametric statistical tests, normality was assessed using the Shapiro–Wilk test, and homogeneity of variance was evaluated using Brown–Forsythe or Levene’s test. For comparisons between two groups, an unpaired two-tailed Student’s t-test was used when parametric assumptions were satisfied. For comparisons among multiple treatment groups, one-way analysis of variance (ANOVA) followed by Dunnett’s multiple comparisons test was used. For experiments involving two independent variables, such as siRNA status and drug treatment, two-way ANOVA followed by Tukey’s or Šídák’s multiple comparisons test was used. When these assumptions were not satisfied, alternative statistical approaches, such as Welch’s ANOVA or non-parametric tests, were considered appropriate. A *p* value < 0.05 was considered statistically significant.

## 5. Conclusions

Narciclasine treatment was associated with reduced HELLS expression, DNA damage-associated signaling, G2/M checkpoint-associated changes, apoptosis, and EMT suppression in CRC cells. The increased sensitivity of HELLS-depleted cells suggests that HELLS may contribute to cellular tolerance against narciclasine-associated DNA damage signaling and apoptosis. These findings support further investigation of HELLS-associated vulnerabilities in CRC cells, while additional rescue experiments will be required to determine whether HELLS downregulation is required for narciclasine-induced anticancer activity.

## Figures and Tables

**Table 1:** Relative UPLC-UV peak-area profile of *Lycoris sanguinea* extract.

No.	Retention Time (min)	Peak Area	Relative UV Peak Area (%)	Remarks
1	1.1	54.8	1.8	
2	1.3	82.41	2.7	
3	1.7	81.49	2.6	
4	2	122.41	4.0	
5	2.6	26.59	0.9	
6	2.8	177.07	5.7	
7	2.9	75.44	2.4	
8	3.2	302.54	9.8	
9	3.3	84.48	2.7	
10	3.4	79.81	2.6	
11	3.5	61.81	2.0	
12	3.6	55.49	1.8	
13	3.7	258.73	8.4	
14	3.9	693.14	22.4	Major detected peak; tentatively annotated as narciclasine
15	6.1	66.32	2.1	
16	6.3	145.63	4.7	
17	7.9	96.62	3.1	
18	13.1	31.98	1.0	
19	13.2	27.04	0.9	
20	13.4	14.15	0.5	
21	13.5	85.27	2.8	
22	13.7	16.15	0.5	
23	13.9	70.87	2.3	
24	14.1	43.62	1.4	
25	15.7	82.11	2.7	
26	15.9	227.55	7.3	
27	16.1	34.15	1.1	

Note: Relative UV peak area (%) was calculated from the UPLC-UV chromatogram at 254 nm and does not represent absolute compound abundance, purity, or weight percentage. Peak 14 was tentatively annotated as narciclasine based on UPLC-QTOF/MS analysis. Absolute quantification was not performed because an authentic narciclasine standard-based calibration curve was not used.

## Data Availability

Publicly available datasets analyzed in this study are available from TCGA/GDC and GEO under accession numbers GSE85871 and GSE196006. Other data are available from the corresponding author upon request.

## Supplementary Materials

The following supporting information can be downloaded at https://www.mdpi.com/article/10.3390/ph19091334/s1. Figure S1. Low-dose narciclasine suppresses wound closure under proliferation-controlled conditions in HT29 and HCT116 cells; Figure S2. Narciclasine increases intracellular ROS-associated fluorescence and activates mitochondria-associated apoptotic signaling in CRC cells; Figure S3. Comparison of basal HELLS expression and narciclasine sensitivity among HIEC-6, HT29, and HCT116 cells; Table S1. Primary antibodies used in this study; Table S2. Primer sequences used for qRT-PCR.

## Abbreviations

CDK	cyclin-dependent kinase
CRC	colorectal cancer
EMT	epithelial–mesenchymal transition
ESI	electrospray ionization
γ-H2AX	phosphorylated histone H2AX
HELLS	helicase lymphoid-specific
ΔΨm	mitochondrial membrane potential
PARP	poly(ADP-ribose) polymerase
siControl	non-targeting control small interfering RNA
siHELLS	HELLS-targeting small interfering RNA
UPLC-QTOF/MS	ultra-performance liquid chromatography–quadrupole time-of-flight mass spectrometry
