# Supplementary material for: Narciclasine Exerts Anticancer Activity in Colorectal Cancer Cells in Association with HELLS Downregulation and DNA Damage-Associated Responses
Source: Pharmaceuticals (Basel). 2026 Aug 24;19(9):1334. doi: 10.3390/ph19091334 (PMC13610751; doi:10.3390/ph19091334)
Supplement: Supplementary file 1 [file pharmaceuticals-19-01334-s001.zip › pharmaceuticals-4433333-supplementary.pdf]

# Supplementary Materials

## Narciclasine Exerts Anticancer Activity in Colorectal Cancer Cells in Association with HELLS Downregulation and DNA Damage-Associated Responses

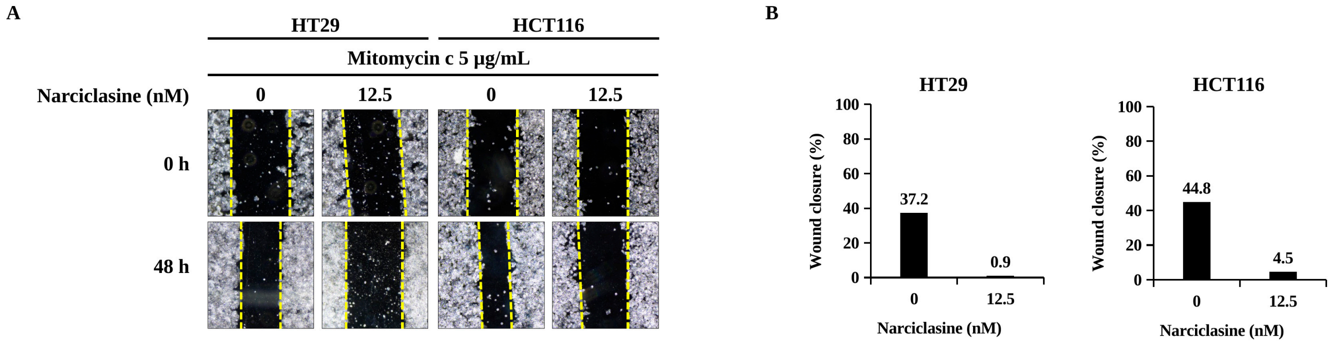


**Supplementary Figure S1.** Low-dose narciclasine suppresses wound closure under proliferation-controlled conditions in HT29 and HCT116 cells. Cells were pretreated with mitomycin C (5 μg/mL) for 2 h and subsequently treated with narciclasine (12.5 nM). Wound images were acquired at 0 and 48 h, and wound closure was quantified. (A) Representative images of wound-healing assays in HT29 and HCT116 cells treated with narciclasine at 12.5 nM for 48 h. To minimize the contribution of cell proliferation, cells were pretreated with mitomycin C at 5 μg/mL for 2 h before narciclasine treatment. Wound images were captured at 0 and 48 h using a microscope with NIS-Elements imaging software (version 4.60). Yellow dashed lines indicate wound boundaries. (B) Quantification of wound closure as a percentage of the initial wound area. Wound closure was calculated using the following formula: [(wound area at 0 h − wound area at 48 h)/wound area at 0 h] × 100.

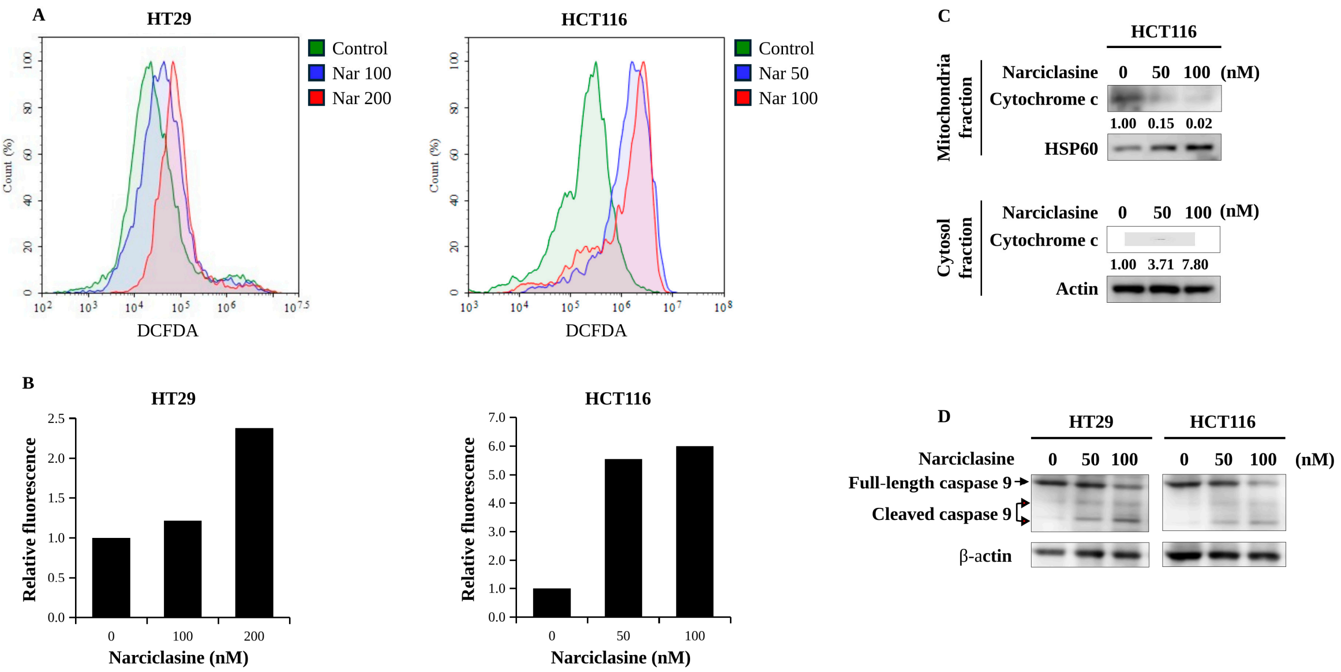


**Supplementary Figure S2.** Narciclasine increases intracellular ROS-associated fluorescence and activates mitochondria-associated apoptotic signaling in CRC cells. CM-H2DCFDA fluorescence was assessed following 24 h of narciclasine treatment, and mitochondrial cytochrome c release and caspase-9 cleavage were examined by immunoblotting. (A) Representative flow cytometric analysis of intracellular ROS-associated CM-$H_2$DCFDA fluorescence in HT29 and HCT116 cells treated with narciclasine for 24 h. HT29 cells were treated with 100 or 200 nM narciclasine, and HCT116 cells were treated with 50 or 100 nM narciclasine. (B) Quantification of relative CM-$H_2$DCFDA fluorescence intensity normalized to untreated control cells. (C) Immunoblot analysis of cytochrome c levels in mitochondrial and cytosolic fractions from HCT116 cells treated with narciclasine for 24 h. HSP60 and β-actin were used as mitochondrial and cytosolic fraction markers, respectively. (D) Immunoblot analysis of full-length and cleaved caspase-9 following narciclasine treatment.

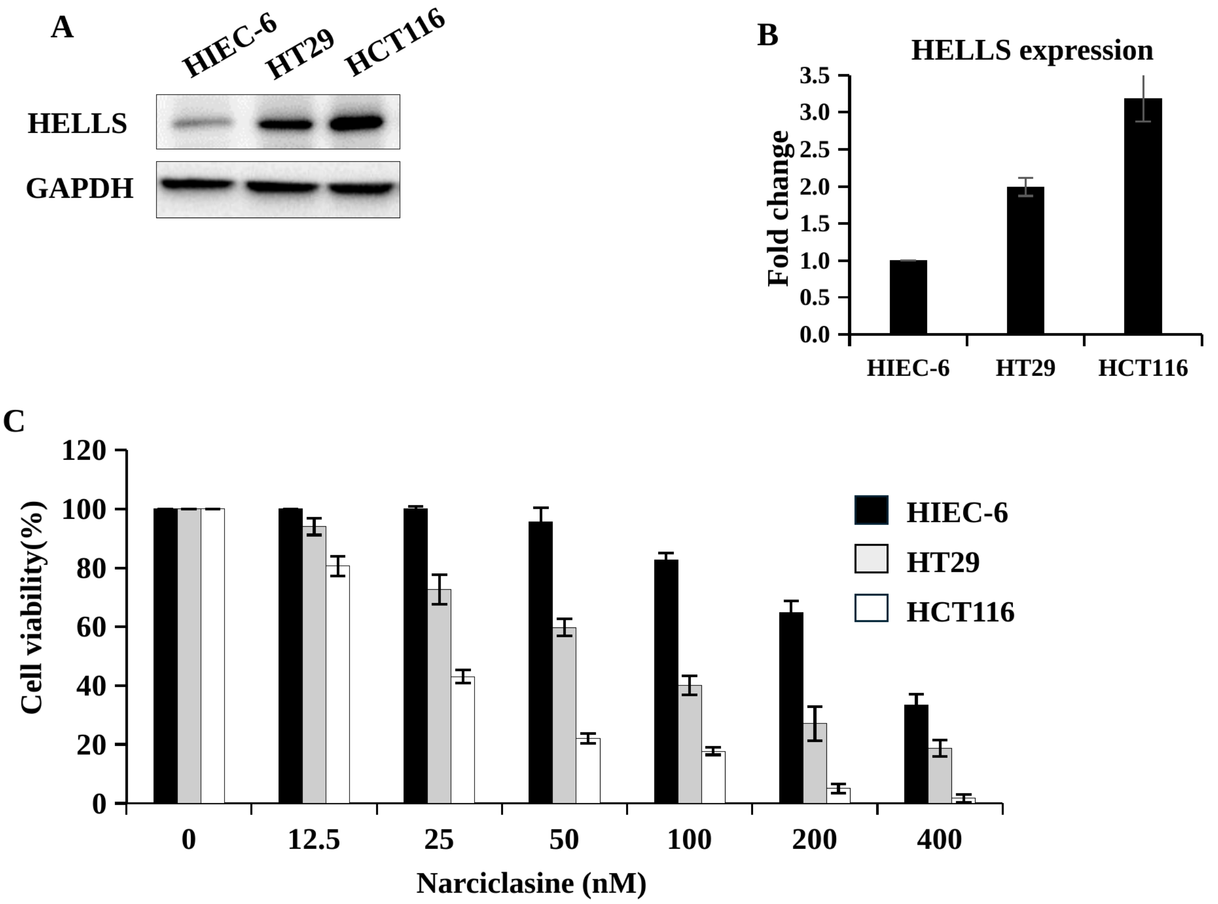


**Supplementary Figure S3.** Comparison of basal HELLS expression and narciclasine sensitivity among HIEC-6, HT29, and HCT116 cells. Basal HELLS expression and cell viability following 24 h of narciclasine treatment were compared between non-malignant HIEC-6 cells and CRC cell lines. (A) Western blot analysis of basal HELLS expression in HIEC-6 non-malignant intestinal epithelial cells and CRC cell lines HT29 and HCT116. GAPDH was used as a loading control. (B) Densitometric quantification of HELLS expression normalized to GAPDH. (C) Cell viability of HIEC-6, HT29, and HCT116 cells treated with increasing concentrations of narciclasine for 24 h, assessed by CCK-8 assay. Data are presented as mean ± SD from three independent experiments.

**Supplementary Table S1.** Primary antibodies used in this study.

| Target | Company | Catalog No. | Dilution |
|---|---|---|---|
| HELLS | Cell signaling Technology | 7988 | 1:3000 |
| γ-H2AX | Cell signaling Technology | 9718 | 1:2000 |
| p53 | Cell signaling Technology | 9282 | 1:1000 |
| p21 | Cell signaling Technology | 2947 | 1:1000 |
| Cyclin B1 | Cell signaling Technology | 4138 | 1:3000 |
| CDK1 | Cell signaling Technology | 9116 | 1:2000 |
| Cyclin A2 | Cell signaling Technology | 4656 | 1:3000 |
| CDK2 | Cell signaling Technology | 2546 | 1:2000 |
| PARP | Cell signaling Technology | 9542 | 1:3000 |
| Bcl-2 | Cell signaling Technology | 2870 | 1:1000 |
| Bad | Cell signaling Technology | 9292 | 1:2000 |
| Bax | Cell signaling Technology | 2772 | 1:2000 |
| Caspase-3 | Cell signaling Technology | 9662 | 1:2000 |
| Cleaved caspase-3 | Cell signaling Technology | 9661 | 1:1000 |
| β-catenin | Cell signaling Technology | 8480 | 1:3000 |
| N-cadherin | Cell signaling Technology | 4061 | 1:1000 |
| E-cadherin | Cell signaling Technology | 3195 | 1:2000 |
| Vimentin | Cell signaling Technology | 5741 | 1:1000 |
| Slug | Cell signaling Technology | 9585 | 1:1000 |
| Snail | Cell signaling Technology | 3879 | 1:1000 |
| β-actin | Cell signaling Technology | 4970 | 1:10000 |
| GAPDH | Cell signaling Technology | 2118 | 1:10000 |
| Cytochrome c | Santa Cruz Biotechnology | sc-13156 | 1:2000 |
| HSP60 | abcam | Ab46798 | 1:2000 |
| Caspase-9 | Cell signaling Technology | 9502 | 1:3000 |
| MUDENG (MuD) | In-house developed | N/A | 1:1000 |

**Supplementary Table S2.** Primer sequences used for qRT-PCR.

| Gene | Forward primer | Reverse primer |
|---|---|---|
| HELLS | GCATGTCTTGGGATAGAGAG | GCTGTTCCATTTTCGTCAAT |
| GAPDH | CCCTCAACGACCACTTTGTC | CCACCACCCTGTTGCTGTA |
